# Rapid establishment of a COVID-19 perinatal biorepository: early lessons from the first 100 women enrolled

**DOI:** 10.1186/s12874-020-01102-y

**Published:** 2020-08-26

**Authors:** Lydia L. Shook, Jessica E. Shui, Adeline A. Boatin, Samantha Devane, Natalie Croul, Lael M. Yonker, Juan D. Matute, Rosiane S. Lima, Muriel Schwinn, Dana Cvrk, Laurel Gardner, Robin Azevedo, Suzanne Stanton, Evan A. Bordt, Laura J. Yockey, Alessio Fasano, Jonathan Z. Li, Xu G. Yu, Anjali J. Kaimal, Paul H. Lerou, Andrea G. Edlow

**Affiliations:** 1 Division of Maternal Fetal Medicine, Department of Obstetrics, Gynecology and Reproductive Biology, Massachusetts General Hospital, 55 Fruit Street, Boston, MA 02114 USA; 2 Division of Neonatology and Newborn Medicine, Department of Pediatrics, Massachusetts General Hospital, Boston, MA USA; 3 Department of Obstetrics, Gynecology and Reproductive Biology, Massachusetts General Hospital, Boston, MA USA; 4 Department of Pediatrics, Massachusetts General Hospital, Boston, MA USA; 5 Department of Pediatrics, Lurie Center for Autism, Massachusetts General Hospital, Boston, MA USA; 6 Vincent Center for Reproductive Biology, Massachusetts General Hospital, Boston, MA USA; 7 Department of Medicine, Brigham and Women’s Hospital, Boston, MA USA; 8 Ragon Institute of the Massachusetts General Hospital, Massachusetts Institute of Technology, and Harvard University, Cambridge, MA USA

**Keywords:** SARS-CoV-2, COVID-19, Pandemic, Biobank, Repository, Pregnancy, Newborn, Vertical transmission, Immune, Obstetrics, Neonatology

## Abstract

**Background:**

Collection of biospecimens is a critical first step to understanding the impact of COVID-19 on pregnant women and newborns - vulnerable populations that are challenging to enroll and at risk of exclusion from research. We describe the establishment of a COVID-19 perinatal biorepository, the unique challenges imposed by the COVID-19 pandemic, and strategies used to overcome them.

**Methods:**

A transdisciplinary approach was developed to maximize the enrollment of pregnant women and their newborns into a COVID-19 prospective cohort and tissue biorepository, established on March 19, 2020 at Massachusetts General Hospital (MGH). The first SARS-CoV-2 positive pregnant woman was enrolled on April 2, and enrollment was expanded to SARS-CoV-2 negative controls on April 20. A unified enrollment strategy with a single consent process for pregnant women and newborns was implemented on May 4. SARS-CoV-2 status was determined by viral detection on RT-PCR of a nasopharyngeal swab. Wide-ranging and pregnancy-specific samples were collected from maternal participants during pregnancy and postpartum. Newborn samples were collected during the initial hospitalization.

**Results:**

Between April 2 and June 9, 100 women and 78 newborns were enrolled in the MGH COVID-19 biorepository. The rate of dyad enrollment and number of samples collected per woman significantly increased after changes to enrollment strategy (from 5 to over 8 dyads/week, *P* < 0.0001, and from 7 to 9 samples, *P* < 0.01). The number of samples collected per woman was higher in SARS-CoV-2 negative than positive women (9 vs 7 samples, *P* = 0.0007). The highest sample yield was for placenta (96%), umbilical cord blood (93%), urine (99%), and maternal blood (91%). The lowest-yield sample types were maternal stool (30%) and breastmilk (22%). Of the 61 delivered women who also enrolled their newborns, fewer women agreed to neonatal blood compared to cord blood (39 vs 58, *P* < 0.0001).

**Conclusions:**

Establishing a COVID-19 perinatal biorepository required patient advocacy, transdisciplinary collaboration and creative solutions to unique challenges. This biorepository is unique in its comprehensive sample collection and the inclusion of a control population. It serves as an important resource for research into the impact of COVID-19 on pregnant women and newborns and provides lessons for future biorepository efforts.

## Background

In December 2019, the newly emerged coronavirus, SARS-CoV-2, led to a surge in cases of pneumonia in Wuhan, China and by March 11, 2020, the World Health Organization classified COVID-19 as a world-wide pandemic. As of July 1, more than 2.6 million people in the United States and 10.6 million worldwide have been diagnosed with COVID-19 [1], with modeling estimates projecting that the percent of the population infected could range from as low as 7% to as high as 70% in the next 5 years [2, 3]. With approximately 140 million live births annually worldwide (https://ourworldindata.org/grapher/births-and-deaths-projected-to-2100), the number of exposed pregnancies could range from the tens to hundreds of millions. Key questions about the impact of COVID-19 infection on the pregnant patient and the developing fetus include, among others: (1) How does pregnancy impact the immune response to COVID-19? (2) What are mechanisms underlying severe maternal morbidity? (3) Does vertical transmission of the virus occur? If so, how? (4) Is breastfeeding safe and does it provide protection for the neonate? (5) What is the impact of maternal immune response to COVID-19 on the developing fetus? The establishment of a COVID-19 biorepository of specimens from the maternal-neonatal dyad is a critical first step to answering these questions; new data suggesting that pregnant women are at increased risk for severe illness from COVID-19 compared to non-pregnant counterparts further demonstrates the importance of research in this unique population [4]. Without the collection and banking of high-quality specimens that enable a full understanding of the immune response and underlying biology, crucial aspects of the impact of COVID-19 on pregnant women and the developing fetus will remain unknown.

Overcoming barriers to the establishment of prospective observational cohort studies during a pandemic requires strategic planning and multidisciplinary effort [5]. Academic medical centers with the ability to leverage available resources, identify shared common goals across stakeholders, and adapt to changing clinical and research needs may be at an advantage [6]. Unfortunately, vulnerable populations such as pregnant women and newborns are at increased risk of exclusion from COVID-19 research and many have advocated on their behalf to be included in key clinical trials and observational studies [7–9]. Moreover the enrollment of pregnant and laboring women on obstetric units presents additional challenges to the timing of and best practices for obtaining ethical and informed consent [10, 11].

Reports concerning the underlying biology of COVID-19 in pregnancy have largely focused on maternal outcomes and the question of vertical transmission [12, 13] and have been limited by their small numbers [14–20] and lack comparison to a control group of uninfected women pregnant during the pandemic. Given the substantial knowledge gaps, and the critical importance of including pregnant women and newborns in COVID-19 research, we sought to establish a biorepository containing wide-ranging sample types from pregnant women and their newborns, with a systematic approach to enrolling pregnant women. Here we describe the unique challenges to enrolling and obtaining samples from the mother-newborn dyad imposed by the COVID-19 pandemic at our institution, and highlight the strategies used to overcome these challenges during the enrollment of the first 100 pregnant women.

## Methods

### Study design

The Partners Institutional Review Board (IRB) approved a prospective, observational cohort study and biorepository of adult patients affected by or at risk for COVID-19 infection at Massachusetts General Hospital (MGH, Partners IRB #2020P000804, approval date March 19, 2020). This study did not exclude pregnant women from enrollment but had no mechanism for enrolling pregnant women systematically, and did not include the collection of critical pregnancy-relevant specimens such as umbilical cord blood, placenta, breastmilk, or vaginal swabs. Due to a healthcare-system-wide mandate that all adult COVID-19 biospecimens research be conducted under the umbrella of the existing protocols, our group wrote an amendment to include the collection of pregnancy-specific specimens, and to enroll the key comparator group of women pregnant during the pandemic but SARS-CoV-2 negative. This amendment was approved on April 20, 2020. Simultaneously, a separate prospective, observational cohort study and biorepository of samples from pediatric patients with COVID-19 infection or at risk of exposure to COVID-19 was established April 1, 2020 (Partners IRB #2020P000955). Due to the healthcare-system-wide mandate prohibiting any additional adult or pediatric biospecimen-related protocols during the height of the pandemic, there were no competing studies during the study period for either adult or pediatric patients.

Universal screening for SARS-CoV-2 in patients presenting to the Labor and Delivery Unit at MGH was initiated on April 16, 2020, due to the high prevalence of COVID-19 in the greater Boston community. Screening and testing in asymptomatic and symptomatic patients occurred via real-time reverse transcription PCR (RT-PCR) for the presence of SARS-CoV-2 on nasopharyngeal swab. CDC criteria were used to define a positive test [21]. Thus, all pregnant women receiving care at MGH after April 20 were eligible for inclusion, and universal screening permitted the inclusion of both SARS-CoV-2 positive women and control women who were asymptomatic and screened negative for SARS-CoV-2 by nasopharyngeal swab within 48 h of admission.

Pregnant women were eligible for inclusion if they met the following criteria: (1) 18 years of age or older, (2) able to provide informed consent or with a healthcare proxy able to do so, (3) diagnosed with, or at risk for SARS-CoV-2 virus infection. As described all participants were tested for the presence of SARS-CoV-2 by RT-PCR of nasopharyngeal swab. Given the study’s primary focus on COVID-19 in pregnancy, contemporaneous controls were enrolled as a convenience sample, from women presenting to Labor and Delivery for care on the same days as enrolled cases. Overnight enrollment was typically restricted to SARS-CoV-2 positive patients, as consent could be obtained only by study clinicians (per hospital policy for SARS-CoV-2-related studies). Newborns born to women who tested positive or negative for COVID-19 were eligible for inclusion in this study. Samples were obtained from newborns in the neonatal intensive care unit (NICU) and the well-baby nursery. The results presented in this paper are reflective of the enrollment of pregnant women and newborns through June 8, 2020. The target enrollment was 200 participants.

### Recruitment

**Fig. 1:**
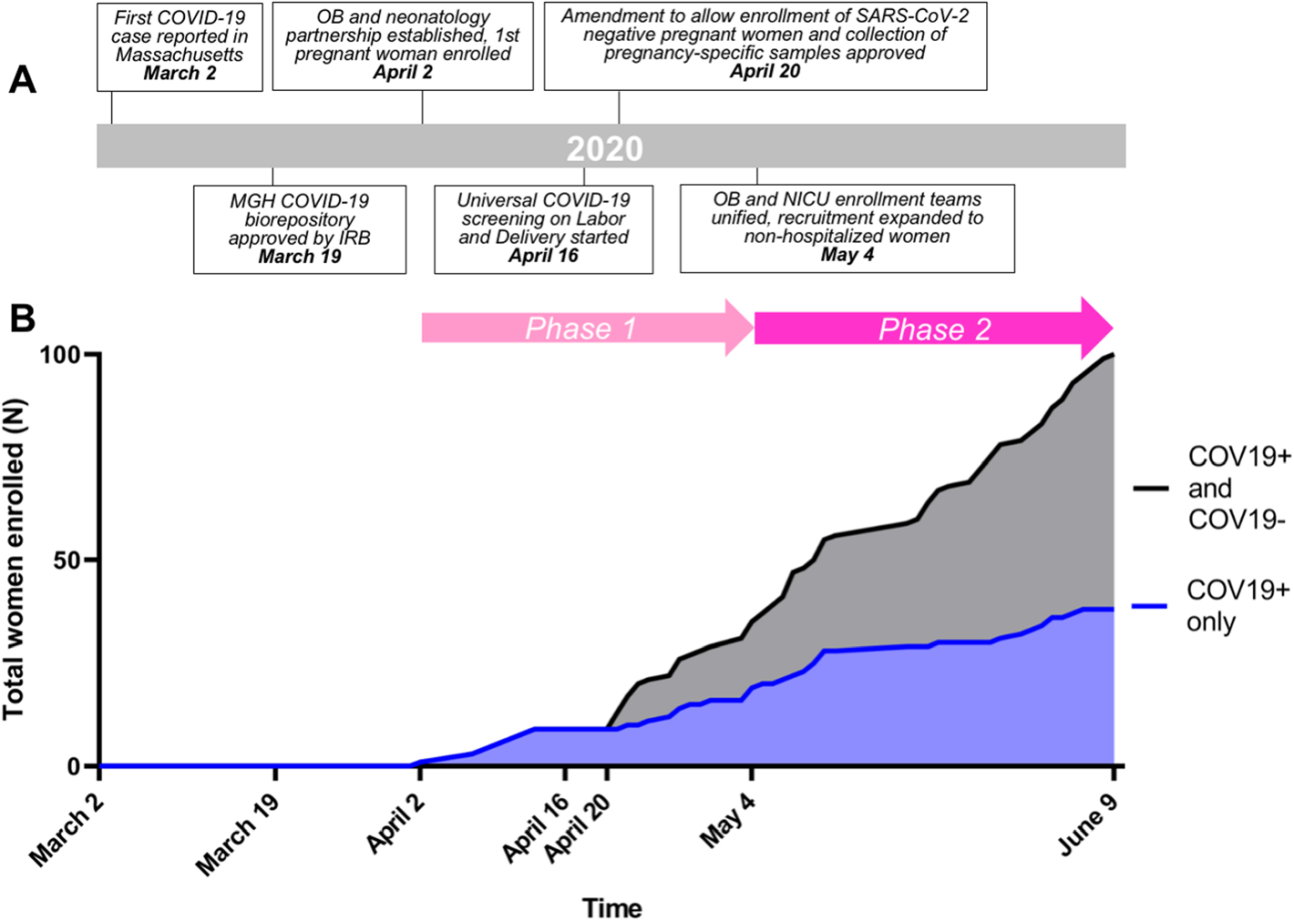
Significant events during establishment of the COVID-19 perinatal biorepository impacting cumulative enrollment of pregnant women over time. **a**. Timeline of events impacting enrollment of pregnant women into the biorepository. **b**. Blue line indicates cumulative enrollment of COV19+ pregnant women. Black line indicates cumulative number of all enrolled pregnant women (COV19+ and COV19-). Phase 1 of enrollment is defined as April 2 to May 4, prior to interventions streamlining enrollment including (1) unification of Obstetrics and Neonatology teams, allowing enrollment into maternal and newborn protocols at the same time and (2) expansion of enrollment efforts to non-hospitalized women. Phase 2 is defined as May 4 to June 9. MGH = Massachusetts General Hospital. COV19+ = mother positive for SARS-CoV-2 on RT-PCR of nasopharyngeal swab at any time during pregnancy; COV19- = mother negative for SARS-CoV-2 on RT-PCR of nasopharyngeal swab when tested for COVID-19 symptoms or as part of universal screening protocol

In the first phase of recruitment (“Phase 1”, Fig. 1b), only inpatients meeting enrollment criteria i.e. pregnant patients hospitalized with COVID-19-related illness or patients presenting to Labor and Delivery for delivery were approached. COVID-19 positive patients were identified in one of the following ways: (1) by dedicated study personnel (obstetrician, Nurse Practitioner or Certified Nurse Midwife) physically stationed on Labor and Delivery enrolling for the study 5–7 days/week; (2) via lists generated in the electronic medical record (EMR) of SARS-CoV-2 positive pregnant patients spanning the inpatient and outpatient setting, including all trimesters; (3) via consults to the antepartum service for pregnant patients with COVID-19 admitted to other services in the hospital (consults are uniformly requested for all off-service pregnant patients with SARS-CoV-2); (4) by automatic COVID-19 flags in the EMR alerting study personnel to patient COVID-19 status on arrival to Labor and Delivery. Contemporaneous SARS-CoV-2 negative controls from the same patient population were approached on the same days for enrollment. Based on the IRB requirements at our institution, the study was first introduced by a member of the clinical team prior to approach by research staff.

All liveborn infants delivered at MGH were eligible for inclusion in the pediatric study. In Phase 1 of recruitment, the obstetric team introduced the pediatric study to the patient, but enrollment of the newborn was performed by the neonatal team at a separate time. Enrollment of newborns occurred either before or after delivery, with the goal of approaching the parents and enrolling the newborn within the first 24 h of life. Due to hospital limitations on research personnel during the pandemic, the initial recruitment team consisted of three obstetricians and two neonatologists. This was expanded to include one certified nurse midwife and one nurse practitioner, and three senior Labor and Delivery nurses. In accordance with hospital-mandated practices developed to conserve personal protective equipment (PPE) and limit exposure of non-essential staff to patients, recruitment was primarily virtual, and occurred by calling the patient’s hospital room. Hospital research restrictions prohibited research assistants or clinical research coordinators from enrolling patients or collecting specimens in the inpatient setting, and virtual phone enrollment was initially limited to clinician investigators (physicians, nurse practitioners, or midwives).

In the second phase of recruitment (“Phase 2”, Fig. 1b), we incorporated two changes to the enrollment process: (1) We added an outpatient recruitment strategy to complement the previously inpatient-only approach. Non-hospitalized, SARS-CoV-2 positive pregnant women receiving care in the unified MGH practice and with due dates within the next 4–6 weeks were virtually approached for inclusion, as were pregnant women scheduled for induction of labor or a planned cesarean section in the upcoming week. (2) We sought to further unify the enrollment strategy and streamline the process for participants by adding Neonatology study staff to the adult study protocol and Obstetrics study staff to the pediatric protocol, so that one study member could consent a participant for both studies before delivery if the patient preferred this. Scripts were developed in both English and Spanish to facilitate a uniform approach to consent by staff on each protocol. Three study staff were fluent in Spanish and there was ready availability of in-person hospital and remote phone Spanish interpreters, facilitating understanding of the study for Spanish-speaking patients who were demonstrated to comprise a substantial proportion of affected patients in our MGH practice [22].

Education of nursing and the Obstetrics and Neonatology staff about the study purpose, eligibility criteria, and specifics of sample collection was conducted via a multipronged approach including: (1) virtual weekly Town Hall meetings; (2) in-person in-services occurring during day and night shifts; (3) training obstetricians in sample collection in a standardized fashion using a brief video created by two of the authors (L.L.S. and A.G.E.) demonstrating best practices for key sample collection on Labor and Delivery; (4) information sheets with step-by-step photo instructions included in the sample collection kits (Additional files 2 and 3), as well as stickers on each individual sample collection container within the kit detailing specifics of collection; (5) fliers posted on Labor and Delivery, the antepartum and postpartum units, the NICU and newborn nurseries (Additional file 4); and (6) research notification flags placed in the electronic medical record of enrolled participants.

### Informed consent and sample use

Participants enrolled in the study provided consent for investigators to access to their electronic medical record, to complete health or symptoms questionnaires, and to collect, process and store samples for COVID-19 related research. For participants who have recovered from COVID-19, the consent form includes the potential for research samples to be collected monthly for 1 year, and every 3 months for 2 years. In addition, participants also consent for potential re-contact for enrollment in future studies. De-identified sample sharing is permitted with approved outside investigators, depending on the nature of request, collaboration with an MGH investigator, and availability of samples. Scientific use of obstetric COVID-19 biorepository samples is determined by the MGH Biospecimens Access Committee, with new experiments requiring a secondary-use IRB. Scientific use of pediatric COVID-19 biorepository samples is determined by the Principal Investigator of the Pediatric IRB protocol (Yonker) in conjunction with a Steering Committee. The adult and pediatric consent forms are provided as Additional files 5 and 6, respectively.

### Sample collection

**Fig. 2:**
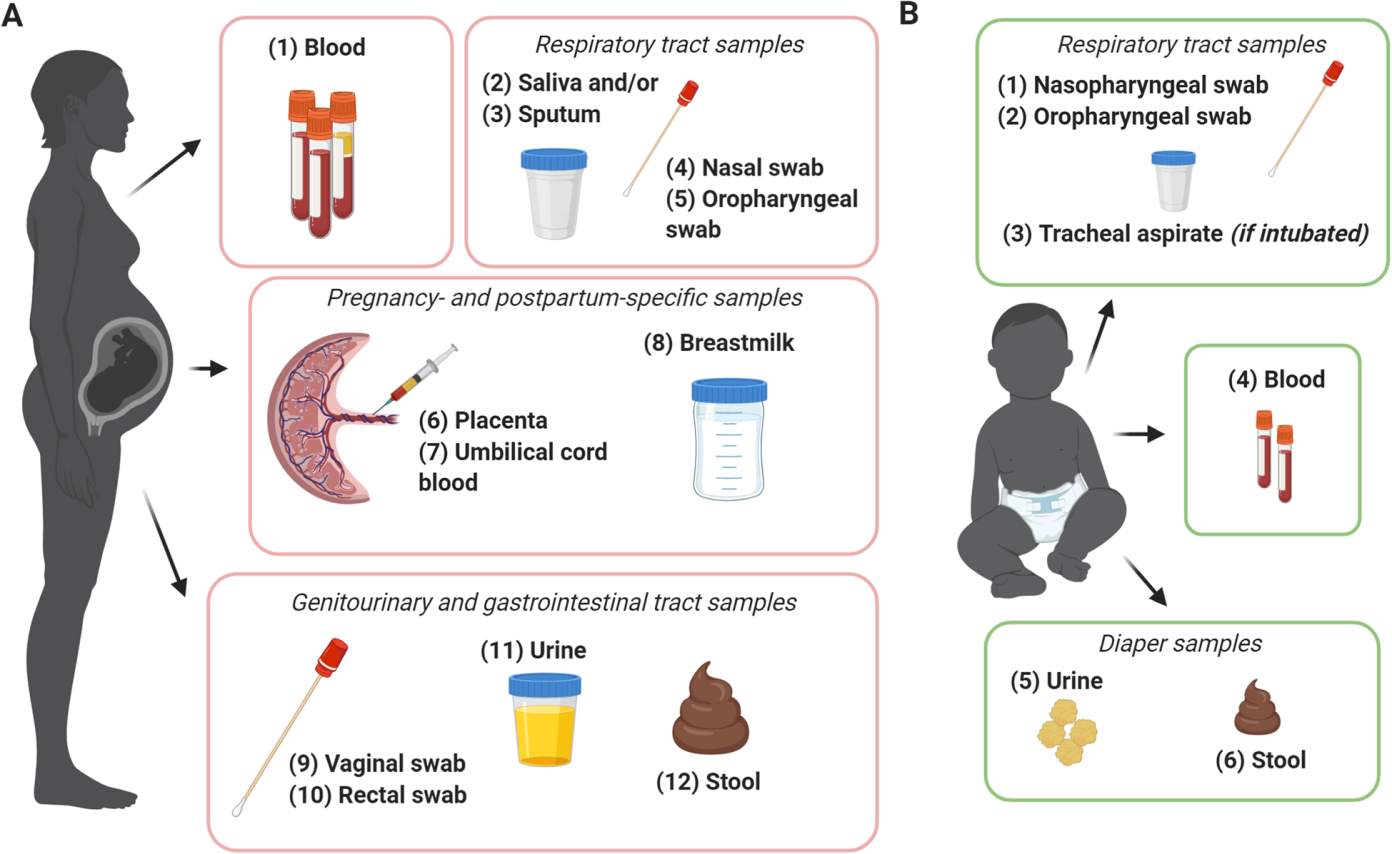
Samples collected on maternal and newborn participants. **a**. Maternal samples included: (1) blood, including 10 mLs in EDTA tubes for plasma, peripheral blood mononuclear cell (PBMC) isolation, and granulocyte or neutrophil isolation; 5–7.5 mL in serum separator tube for serum; 2.5 mL in PaxGene tube for RNA; (2) saliva and/or (3) sputum (sputum if patient had productive cough); (4) nasal swab; (5) oropharyngeal swab; (6) maternal and fetal side placental biopsies for RNA extraction, and formalin-fixed paraffin-embedded full thickness placental block for in situ placental histopathology; (7) umbilical cord blood (EDTA, serum separator tube and PaxGene as described above for maternal blood); (8) colostrum or mature breastmilk; (9) vaginal swab; (10) rectal swab; (11) urine; (12) stool. Maternal blood was preferentially collected during a clinical blood draw by the clinical nurse. Placental biopsies and cord blood were collected by the obstetrical care team immediately after delivery, with support from study staff when possible. Women who planned to breastfeed were encouraged to clean the breast per instructions and self-collect any amount of colostrum or mature milk prior to discharge from the hospital. **b**. Newborn samples included: (1) nasopharyngeal swab; (2) oropharyngeal swab; (3) tracheal aspirate (if relevant); (4) neonatal blood collected into EDTA microtainer via heel-stick with clinical metabolic screen at 24–36 h of life; (5) urine collected using cotton balls placed into diaper; and (6) stool. Figure created with BioRender.com and reproduced with permission

Once a participant was enrolled, samples could be collected at all or any of three timepoints: antenatal, during the delivery hospitalization, or up to 12 weeks postpartum. In addition, for recovered participants, samples could be collected at regular intervals for up to 2 years after enrollment. Participants could elect to provide all samples or could decline to provide specific samples. Mothers gave separate informed consent for their newborn to participate in the pediatric study. Maternal specimens were collected primarily by Obstetrics study team members upon enrollment or by the patient’s clinical team if enrollment or delivery occurred overnight. Sample collection bags were pre-assembled into kits and labeled with randomly-generated patient identifiers. Upon enrollment, patients were assigned a study kit, which were kept on Labor and Delivery. Collection kit components are listed in Additional file 1, Table S1 and kit assembly instructions are provided in Additional file 7. Weekly video-conference Town Hall meetings and focused emails to clinicians were used to provide reminders and updates about the study to ensure adherence to best practices and ongoing provider awareness of the study. Samples collected from maternal and newborn subjects are depicted in Figure 2. Processing and storage characteristics of collected samples are detailed in Table 1.

**Table 1:** Sample type, processing details and storage characteristics of maternal and neonatal specimens

Sample Type	Processing details	Storage characteristics
Maternal blood^a^ ● SST × 1 ● EDTA × 2 ● PaxGene × 1 Umbilical cord blood^a^ ● SST × 1 ● EDTA tube × 2 ● PaxGene tube × 1 Neonatal blood^b^ ● EDTA microtainer × 2	Serum SST tube spun at 1200 g × 10 min at RT, aliquoted into cryovials Plasma EDTA tube spun at 1000 g × 10 min at RT, aliquoted in cryovials Ficoll-based PBMC isolation Following plasma removal, remainder of blood diluted with 1:1 HBSS, layered on top of Ficoll at 2:1 ratio, spun at 1000 g at 30 min; PBMC layer collected, diluted in HBSS for counting Granulocyte/neutrophil isolation Isolated using EasySep Direct Human Neutrophil Isolation Kit (StemCell Technologies) for subsequent RNA or DNA analysis PaxGene Shaken vigorously at time of collection, store at RT for at least 2 h	Serum − 80 °C Plasma − 80 °C PBMCs Suspended in freezing medium at − 80 °C × 24 h, then transferred to liquid nitrogen for long term storage Granulocytes/neutrophils RNA: Resuspended at 1 × 105 cells/tube in Buffer TCL (Qiagen) and 1% BME DNA: Pelleted at 5 × 106 cells/tube and stored PaxGene Collection tube stored in − 20 °C overnight, then transferred to − 80 °C
Saliva^a^ Sputum^a^	Mixed 1:1 with DTT and aliquoted	−80 °C
Nasal swab^a^ Nasopharyngeal swab^b^ Oropharyngeal swab^a, b^	Collected in PBS, stored immediately at 4 °C after collection, processed within 3–4 h; PBS collection media aliquoted	−80 °C
Vaginal swab^a^ Rectal swab^a^	Stored immediately at 4 °C after collection; stalk removed, swab tip placed in cryovial and stored within 3–4 h of collection	−80 °C
Urine^a, b^	Maternal urine aliquoted into cryovials Neonatal urine-soaked cotton balls from newborns transferred to 60 mL syringe to dispense urine; aliquoted into cryovials	−80 °C
Placenta^a^	Biopsies for RNA analysis 2 x 5mm^3^ biopsies collected from maternal side and fetal side immediately after delivery, washed in PBS × 2, stored upright in RNAlater at 4 °C × 24 h. Sections for histopathology Full thickness placental sections fixed in formalin and paraffin-embedded into blocks for in situ histopathology	Biopsies for RNA analysis Biopsies divided into ~ 50 mg pieces and snap frozen in liquid nitrogen, then stored at − 80 °C Sections for histopathology RT
Stool^a, b^	Microspatula used to dispense ~ 1 cc into 1 mL RNAlater, empty cryovials, or 1 mL Buffered Glycerol Saline (Fisher)	−80 °C
Breastmilk^a^	Stored at 4 °C after collection, milk collection tube rewarmed in hands briefly emulsify lipids then aliquoted into cryovials within 3–4 h of collection	−80 °C
Tracheal aspirates^b^	Resuspended in trizol and aliquoted into cryovials	−80 °C

*SST* Serum separator tube, *PBMC* Peripheral blood mononuclear cells, *HBSS* Hank’s balanced salt solution, *BME* β-mercaptoethanol, *DTT* DL-Dithiothreitol, *RT* room temperature. ^a^maternal sample. ^b^neonatal sample

### Cost

The per patient cost of the study was approximately $120 for consumables related to sample collection kits and processing. This cost did not include personnel salary costs. The most expensive components of specimen collection kits included PaxGene tubes, RNALater, and stool/urine hats. Foregoing blood collection in PaxGene tubes for later RNA sequencing and taking fewer placental biopsies to minimize RNALater use could be ways to reduce per-patient costs. The most expensive components of sample processing included the cost of personal protective equipment for laboratory processors, the cost of Ficoll and other reagents related to blood processing and PBMC (peripheral blood mononuclear cell) isolation, and printable freezer-safe labels and cryovials for long-term specimen storage. Hand-labeled tubes and less expensive storage vials might be an area in which cost-savings could be achieved. Of note, many expensive, necessary, and back-ordered supplies were generously donated by research laboratories throughout MGH in an effort to support the establishment of this biorepository during a critical window of time.

### Sample annotation

Samples were extensively annotated using REDCap electronic data capture tools hosted at MGH [23]. Data were hand abstracted from the electronic medical record by study clinical research coordinators (CRCs) trained by an obstetrician (A.A.B.). The first five abstracted charts for each CRC were reviewed by the principal investigator (A.G.E.). One in ten charts was subsequently reviewed for quality control. REDCap data fields are depicted in Additional file 8.

### Statistical analysis

Simple linear regression models were used to predict the rate at which women were enrolled over time, and the differences in slopes during Phase 1 versus Phase 2 were compared. Demographic characteristics of the enrolled cohort were compared to the overall MGH delivery population during the study period using Mann-Whitney or Chi-square tests where appropriate. The median number of samples collected per maternal participant was calculated before and after key enrollment strategy changes, and for SARS-CoV-2 positive and SARS-CoV-2 negative groups. Differences between groups were assessed by the Mann-Whitney test. The proportion of women or newborns in which each sample type was collected were compared between SARS-CoV-2 positive and SARS-CoV-2 negative groups by Fisher’s exact test. For all analyses, *P* < 0.05 was considered statistically significant. Analyses were performed using GraphPad Prism (v8, San Diego, CA).

## Results

### Description of enrollment

**Fig. 3:**
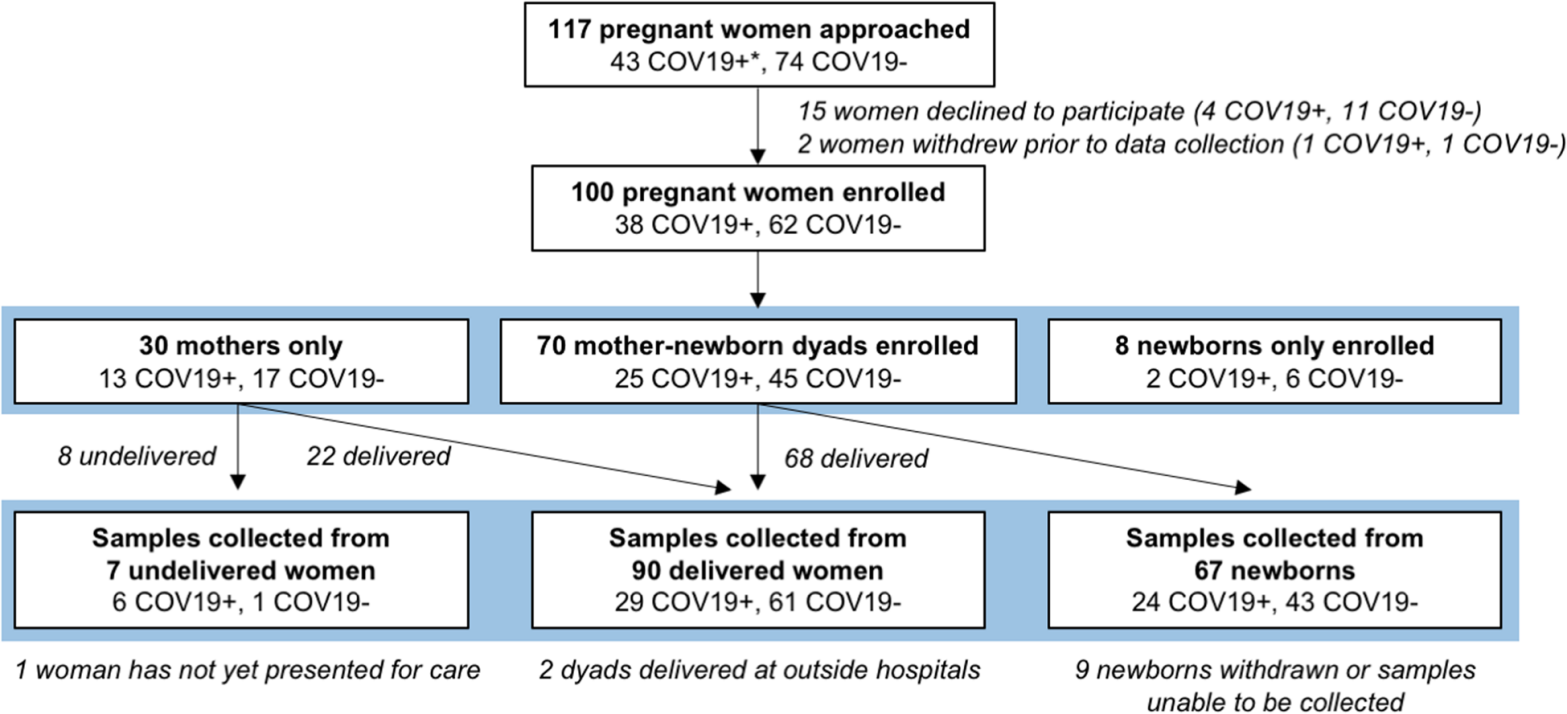
Flow chart of participant enrollment and cohort in which samples have been collected. *Nine patients admitted to MGH who tested positive for SARS-CoV-2 were not approached for enrollment. Five of nine delivered precipitously and could not be enrolled prior to delivery, and four of nine were admitted and delivered during time periods when study staff were not available to consent the patient. COV19+ = mother positive for SARS-CoV-2 on RT-PCR of nasopharyngeal swab at any time during pregnancy; COV19- = mother negative for SARS-CoV-2 on RT-PCR of nasopharyngeal swab when tested for COVID-19 symptoms or as part of universal screening protocol

A flow chart describing study participants is presented in Fig. 3. Of the 117 pregnant women approached for enrollment between April 2 to June 9, 100 women and 78 newborns were enrolled in the biorepository. Of the first 100 women enrolled in the study, 38 were SARS-CoV-2 positive and 62 were SARS-CoV-2 negative at enrollment. Women were considered “SARS-CoV-2 positive” if they tested positive for SARS-CoV-2 at any time during their pregnancy by a clinical nasopharyngeal RT-PCR test. Time from symptom onset and positive test to collection of each specimen is documented in the REDCap sample annotation. Enrolled participants include asymptomatic women who screened positive for SARS-CoV-2 through universal testing (*n* = 15); symptomatic women who tested positive (*n* = 23); symptomatic women who tested negative (*n* = 8); and asymptomatic women who screened negative (*n* = 54). Newborns were categorized by their mother’s SARS-CoV-2 status, as determined by these criteria. Thirty enrolled women declined newborn participation, and 8 women enrolled remote from delivery have not yet decided on newborn enrollment.

Prior to the direct involvement of the Obstetrics and Neonatology team on April 2, no pregnant women had been enrolled in the study. The cumulative number of enrolled pregnant women over time increased significantly after passage of the amendment allowing enrollment of all pregnant women at risk for COVID-19 and collection of pregnancy-specific samples (placenta, umbilical cord blood, vaginal swabs, and breastmilk), indicated in Fig. 1. Prior to this amendment, 10 out of 10 enrolled participants were COVID-19 positive; after SARS-CoV-2 negative controls were no longer excluded, this proportion decreased significantly to 38/100 (*P* = < 0.0001). Contrary to expectations, the rate at which enrolled mothers chose to enroll their newborn in the pediatric study during Phase 1 compared to Phase 2 did not increase (74% vs 77%, *P* = 0.79). However, the overall rate at which dyads were enrolled in the biorepository did increase from 5 dyads per week to over 8 dyads per week (*P* < 0.0001) from Phase 1 to Phase 2, reflective of the improved overall efficiency of the enrollment process.

### Generalizability and representativeness of the cohort

Demographic information for the first 100 participants enrolled in the COVID-19 perinatal biorepository cohort compared to the overall MGH delivery population during the study period are presented in Table 2. Compared to the general Labor and Delivery population at MGH, women enrolled in the biorepository were more likely to be Hispanic, publicly-insured, and Spanish-speaking. These demographics are consistent with our prior work demonstrating that pregnant women of Hispanic ethnicity who receive prenatal care at MGH community health centers have been disproportionately impacted by COVID-19 [22].

**Table 2:** Demographic characteristics of women enrolled in COVID-19 perinatal biorepository compared to women delivering at MGH during study period

	COVID-19 biorepository (***N*** = 100)	MGH labor and delivery population (***N*** = 736)	*P*-value
**Maternal Age**^**a**^	33 (29, 37)	33 (30, 36)	0.95
**Race**			< 0.0001
*Asian*	4 (4%)	88 (12%)	
*Black/African American*	6 (6%)	52 (7%)	
*White*	59 (59%)	459 (62%)	
*Other*	18 (18%)	124 (17%)	
*Unknown/not reported*	13 (13%)	13 (2%)	
**Ethnicity**			< 0.0001
*Hispanic or Latina*	40 (40%)	128 (17%)	
*Not Hispanic or Latina*	54 (54%)	578 (79%)	
*Unknown/Not Reported*	6 (6%)	30 (4%)	
**Type of Insurance**			< 0.0001
*Private*	57 (57%)	573 (78%)	
*Public*	42 (42%)	155 (21%)	
*Other*	1 (1%)	8 (1%)	
**Primary Language**			< 0.0001
*English*	67 (67%)	614 (83%)	
*Spanish*	29 (29%)	68 (9%)	
*Other*	4 (4%)	51 (7%)	
*Missing*	0 (0%)	3 (0.4%)	

^a^Presented as median (IQR)

### Description of samples collected

**Fig. 4:**
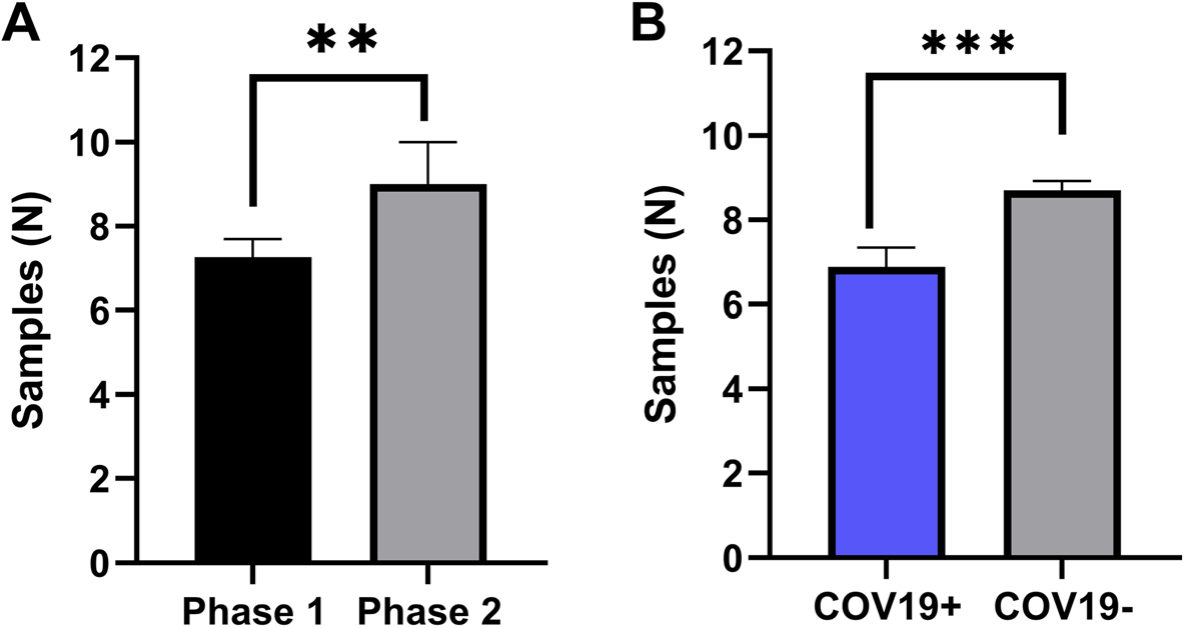
Total number of samples collected on each maternal participant by phase of enrollment and by SARS-Cov-2 status. **a**. Mean number of samples collected on each maternal participant was greater from women enrolled during Phase 2 compared to Phase 1. **b**. Mean number of samples collected per maternal participant was greater in SARS-CoV-2 negative than positive women. Data depicted as mean +/− SEM, **P < 0.01,***P < 0.001

Women were eligible to give 12 different sample types during their delivery hospitalization; undelivered women could provide 9 (all sample types except for placenta, umbilical cord blood, and breastmilk). Compared to Phase 1, significantly more samples were successfully collected from participants during Phase 2 (7 vs 9 samples per participant, *P* = 0.004, Fig. 4a). The median total number of samples collected per maternal participant was higher in women who were SARS-CoV-2 negative than positive (9 vs 7 samples, *P* = 0.0007, Fig. 4b).

The highest sample yields were for placenta (96%), umbilical cord blood (93%), urine (99%), and maternal blood (91%). The least-donated sample types were maternal stool (30%) and breastmilk (22%). Of the 61 enrolled women who delivered enrolled newborns, significantly fewer women agreed to neonatal blood sampling, compared to maternal blood (39 vs 57, *P* = 0.0001) or umbilical cord blood (39 vs 58, *P* < 0.0001).

**Fig. 5:**
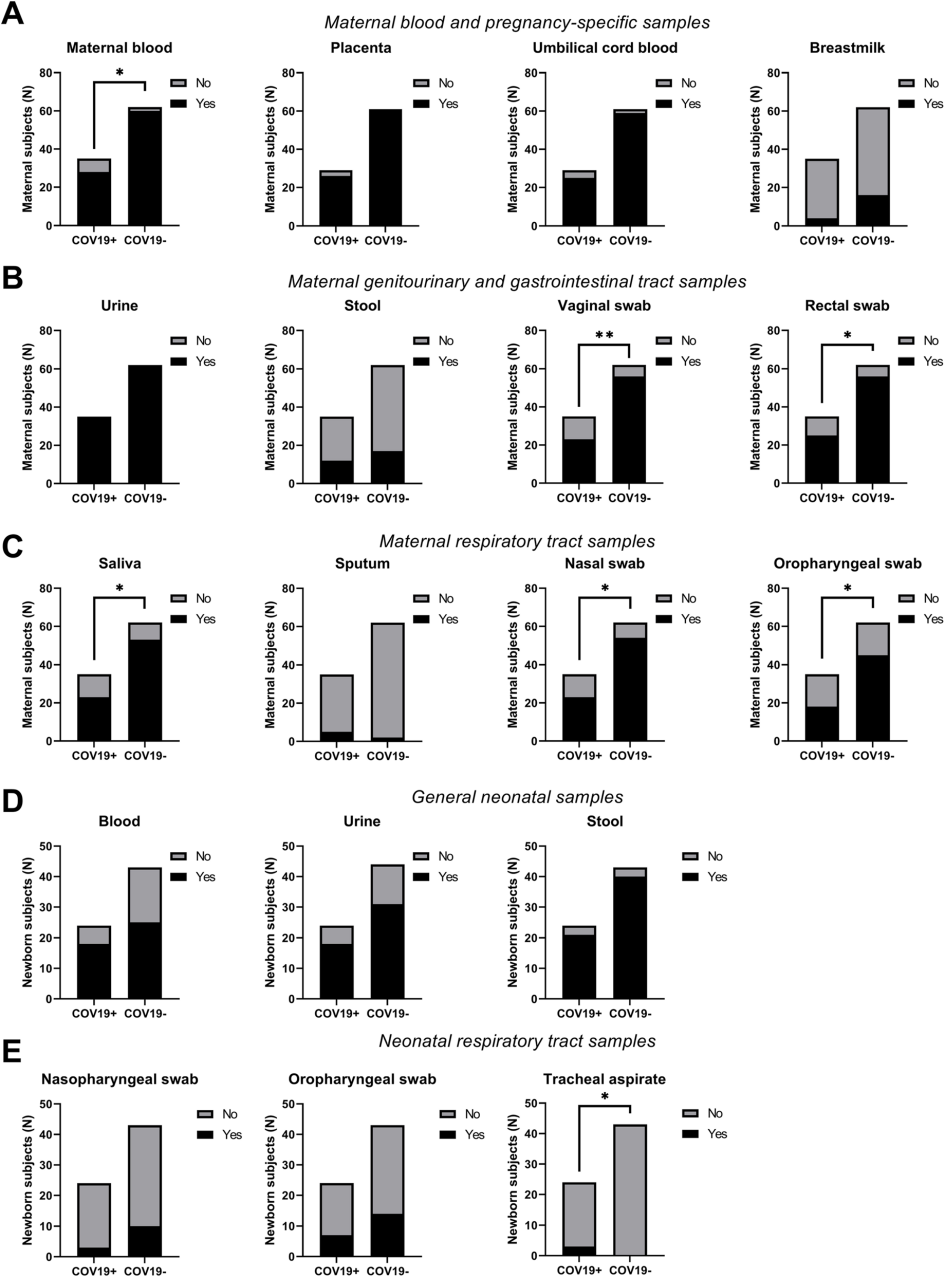
Proportion of samples collected from maternal and newborn participants by COVID status. **a**. Proportion of women in which maternal blood, placenta, umbilical cord blood, and breastmilk were collected, by COVID status. **b**. Proportion of women in which urine, stool, vaginal swabs, and rectal swabs were collected, by COVID status. **c**. Proportion of women in which saliva, sputum, nasal swabs, and oropharyngeal swabs were collected, by COVID status. **d**. Proportion of newborns in which blood urine and stool were collected by mother’s COVID status. **e**. Proportion of newborns in which nasopharyngeal, oropharyngeal, and tracheal aspirate samples were collected by mother’s COVID status. COV19+ = mother positive for SARS-CoV-2 on RT-PCR of nasopharyngeal swab at any time during pregnancy; COV19- = mother negative for SARS-CoV-2 on RT-PCR of nasopharyngeal swab when tested for COVID-19 symptoms or as part of universal screening protocol. *P < 0.05, **P < 0.01. The number of maternal and newborn participants providing each sample type by COVID status is presented in Additional file 1, Tables S2 and S3, respectively

Figure 5 depicts the proportion of women and newborns providing each sample type by SARS CoV-2 status. Maternal blood, saliva, nasal swabs, oropharyngeal swabs, vaginal swabs, and rectal swabs were more often collected in SARS-CoV-2 negative than SARS-CoV-2 positive women. Tracheal aspirates were collected in 3 intubated newborns, all of whom were born to SARS-CoV-2 positive mothers, and intubated due to complications of prematurity. No newborns born to SARS-CoV-2 negative mothers required intubation. The number of maternal and newborn participants providing each sample type by SARS-CoV-2 status is presented in Additional file 1, Tables S2 and S3, respectively.

## Discussion

During the initial phase of the COVID-19 pandemic in Massachusetts, guided by the state Department of Public Health [24], institution-wide policies were enacted at MGH that dramatically impacted both clinical care and research activities. These policies included limitations on conducting research unrelated to the COVID-19 pandemic, redeployment of staff to COVID-19 units, social distancing procedures limiting the number of on-site hospital employees, and limiting face-to-face encounters with COVID-19 patients to essential providers to conserve PPE. The establishment of COVID-19 adult and pediatric biorepositories was made possible by a centralized effort to quickly facilitate the collection of data and samples during the peak of the pandemic in Boston. Importantly, although pregnant women and their newborns were not excluded from the original iterations of the adult and pediatric biorepositories, there was not yet a mechanism or defined strategy to facilitate enrollment and sample collection in these unique populations. Between March 19 and April 2, prior to the involvement of the Obstetrics and Neonatology services, no pregnant women or newborns had been enrolled in the biorepository.

The Obstetrics and Neonatology team worked together with the Principal Investigators of the existing adult (Yu, Li) and pediatric (Yonker) protocols to ensure the successful inclusion of pregnant women and their newborns. An amendment was submitted to the adult IRB protocol to include the collection of pregnancy-specific samples, and a team of Obstetrics and Neonatology clinicians trained to both enroll and collect patient samples was assembled. The first pregnant SARS-CoV-2 positive woman was enrolled on April 2. Between April 2 and April 20, ten symptomatic, SARS-CoV-2 positive women and the majority of their newborns (for those who delivered) were enrolled in the biorepository. After the amendment was passed on April 20 permitting collection of pregnancy-specific samples (vaginal swab, placenta, cord blood and breastmilk) and enrollment of all pregnant women at risk for COVID-19, including a control population of women screening negative for SARS-CoV-2 on hospital admission, enrollment increased substantially, as demonstrated in Fig. 1.

Compared to the general adult or pediatric populations, enrollment of pregnant or newly postpartum women and their newborns requires the clinical expertise and creative input of both the Obstetrics and Neonatology study teams. We found that most pregnant women approached for enrollment were experiencing added emotional stressors – including fear of testing positive during universal screening, fear of exposing themselves or their newborn to COVID-19 during the delivery admission, and anxiety over the unknown impact of a COVID-19 diagnosis on their pregnancy and newborn. The time, effort, and sensitivity required to discuss participation in COVID-19 research during an inherently stressful hospitalization – whether for COVID-19 illness or for labor and delivery – was significant, and there were time constraints inherent in obtaining consent as we hoped to consent women for the study before the initial clinical blood draw or IV placement to facilitate research sample collection for those participants who wished to give blood. In addition, the mandate for virtual rather than in-person consent, due to strict limitations on face-to-face encounters to protect patients and staff and conserve PPE, posed another challenge to consenting patients in a dynamic and sometimes stressful situation. These challenges further exacerbated the ethical conundrum around enrolling women in labor or in the time-period around obstetric challenges, when pain, anxiety, and stress due to the normal physiology of labor or an obstetric complication may complicate the recruitment and consent process [10, 25].

It became clear that a second conversation with Neonatology to enroll the newborn added to logistical challenges and time burden for participants, study staff, and clinical care providers. Due to these observations, a unified Obstetrics/Neonatology enrollment strategy was initiated (Phase 2). The rate at which mother-newborn dyads were enrolled increased from 5 per week to over 8 per week after this change, and our sample collection yield increased significantly after this intervention as well. These data suggest that future efforts to establish pregnancy biorepositories with an aim of capturing the dyad would be best served by having a single IRB protocol encompassing both maternal and newborn populations, permitting a single consent form and approach- this was not possible in our case due to hospital mandates limiting new COVID-19-related research protocols.

Cross-training both study teams on both the adult and pediatric protocols, developing scripts for the consent to standardize the process, and instituting an outpatient consent strategy to complement the inpatient enrollment improved overall enrollment and sample collection rates. Although women were not more likely to agree to newborn participation after this strategy was implemented, the rate at which mother-newborn dyads were enrolled increased overall, from 5 per week to over 8 per week. The increase in number of maternal samples collected per patient after the initiation of a collaborative, unified enrollment strategy reflects the ability of Obstetrics study team members to focus on facilitating maternal sample collection, particularly of time-sensitive maternal blood and delivery specimens. Improvements in collection procedures, familiarity and willingness of clinical team members to assist in sample collection, and pre-identification of women undergoing planned deliveries by cesarean section or induction of labor for enrollment to facilitate sample collection during daytime hours all likely contributed to higher rates of sample collection over time.

Our data indicate that more samples overall were collected from SARS-CoV-2 negative than positive women. As SARS-CoV-2 negative participants were approached during daytime hours and were often undergoing scheduled induction of labor or cesarean delivery, the more predictable timing may have facilitated sample collection as well as participant willingness to donate particular samples for research. In contrast, many SARS-CoV-2 positive participants were enrolled after arriving to Labor and Delivery in labor or after an unexpected screen positive on universal screening during admission for delivery. The inherently more unpredictable nature of these admissions and events may have contributed both to reduced ability of study clinicians to obtain time sensitive samples prior to delivery (such as vaginal and rectal swabs which were less reliable when contaminated by blood after delivery), and reduced willingness of participants to provide certain samples in the setting of emotional stress. Supporting the latter point, we found respiratory samples were also more often collected from SARS-CoV-2 negative women. As these samples can be collected at any point during hospitalization and were less likely to be impacted by study staff availability or timing of delivery, differences in sample retrieval are more likely reflective of the subject’s willingness to provide these samples. In addition to possible increased stress of SARS-Co-V-2 positive participants, which is an important area for future study, there are several other potential explanations for reduced sample collection from SARS-CoV-2 positive participants. SARS-CoV-2 positive women may have had more fear of discomfort associated with the nasal and oropharyngeal sample collections, as many of these women had to undergo more than one clinical nasopharyngeal swab for SARS-CoV-2 prior to their enrollment. It is also possible that despite appropriate availability and training on usage of PPE, study staff discomfort with obtaining respiratory samples on known SARS-CoV-2 positive women may have contributed to decreased collection rates.

Delivery specimens (placenta, umbilical cord blood) were successfully collected on greater than 90% of women who delivered. Strategies that likely contributed to this high collection rate included outpatient enrollment of women presenting for timed deliveries (induction of labor or scheduled cesarean sections), providing in person in-services, as well as written and video instructions on sample collection procedures to the clinical teams, and recruitment of senior Labor and Delivery nurses to the study team to assist with intrapartum and postpartum sample collection. We noted low rates of collection of stool and breastmilk, possibly due to early maternal discharge protocols during the pandemic, which limited the number of women who had full return of bowel function and established breastmilk supply prior to discharge.

Newborn sample collection rates were highest for stool and urine, specimens considered clinical waste that required no additional procedure for the newborn. Urine was initially collected via urine bag, which is a standard, non-invasive method to collect urine from newborns. Several parents were distressed by the urine bag adhesive on newborn skin or expressed frustration with urine leakage. We therefore changed to collecting urine-soaked cotton balls placed in the diaper. While extracting urine from the cotton balls was slightly more challenging, this change improved parental and nursing satisfaction with the study. Maternal consent for a research-only, neonatal venous blood draw was significantly lower than consent given for maternal blood draw or draw from the umbilical cord. One strategy that reduced parental discomfort with neonatal blood draw was to instead collect an additional small volume of blood from the heel-stick used for the metabolic screen performed on every newborn in the state of Massachusetts, thus eliminating the need for an additional/clinically unnecessary procedure.

The reallocation of resources to the care of adult COVID-19 patients in our hospital included deploying pediatric respiratory therapists and NICU ventilators to the adult intensive care unit, and transferring out all newborns who required ventilatory support to other hospitals. This in turn affected admission of high risk and extremely preterm newborns. Furthermore, there was an unanticipated backorder of nasopharyngeal swabs from April 29 to May 12, likely due to increased clinical demand. Thus, both the opportunity and ability to obtain tracheal and nasopharyngeal neonatal samples were limited during this enrollment period.

Strengths of our study include the prospective enrollment of both COVID-19 positive pregnant women and controls who were asymptomatic and SARS-CoV-2 negative by nasopharyngeal swab. Both cases and controls delivered during the peak of the COVID-19 pandemic in Massachusetts. Such controls permit the most accurate assessment of the impact of SARS-CoV-2 infection, versus maternal stress and other unmeasured factors, on pregnancy and neonatal outcomes and biological tissue and fluid changes during the pandemic. We enrolled pregnant women across the full spectrum of disease severity, from critically ill to asymptomatic. We successfully recruited and enrolled 38 SARS-CoV-2 positive women and 25 of their newborns in a narrow time frame, overcoming major institutional changes that impacted the delivery of clinical care and research activities across all departments. Strengths of our methodology in establishing this biorepository include the synergistic efforts between Obstetric and Neonatology departments founded on a shared desire to advocate for inclusion of our unique patient populations. We describe a combined maternal and newborn consent process that increased the rate of recruitment, allowed for efficient use of time and resources, and likely improved the experience of women approached for enrollment (an important area for future study). High rates of collection of matched maternal blood, placenta, umbilical cord blood, neonatal blood, and neonatal specimens from enrolled participants is also a strength. While initial sample collection in acutely ill and convalescent pregnant and postpartum participants is the focus of this manuscript describing the earliest biorepository efforts, the potential for serial sample collection for up to 2 years after recovery in this cohort will permit assessment of the risk of reinfection and potential duration of protection in a dyad, also a strength of our study methodology.

Our study is not without limitations. Our experience in setting up a maternal-newborn biorepository was limited by state- and institution-wide mandates imposing restrictions on the activities of clinical and research personnel. Our ability to collect certain samples, particularly newborn nasopharyngeal swabs and maternal vaginal swabs, was significantly impacted by a national shortage of needed swab types during March and April. Although we experienced shortages of collection, processing and storage supplies, generous donations from other investigators whose research activities had been suspended allowed us to continue enrollment and sample collection. While the samples in the perinatal biorepository are annotated with clinical outcomes through delivery and the postpartum period, longer-term follow-up studies of both the maternal and pediatric cohorts are planned in collaboration with Pediatric, Cardiology, Psychiatry, and Neurology colleagues. These long-term outcomes can in turn be correlated with perinatal biology, providing the potential to enrich understanding of the biological basis for long-term outcomes after COVID-19.

Although other institutions and collaboratives in the United States and internationally are developing COVID-19 pregnancy registries with (e.g. the PRIORITY study) [26] and without (e.g. IRCEP or the International Registry of Coronavirus in Pregnancy) perinatal specimen biorepositories, to our knowledge, the scope and size of these COVID-19 pregnancy-related biorepositories have not yet been published. Some national and international perinatal biorepositories with a broader and non-disease-specific focus have published enrollment numbers that are substantially larger than our perinatal biorepository (e.g., over 9000 women enrolled in the Generation R Study Biobank and over 10,000 women in the PeriBank) [27, 28]. These repositories differ from ours, in that they are not limited to studying the effects of a single defined exposure, and enrollment has taken place over many years.

Data from other institutions in areas highly impacted by COVID-19, such as New York City and urban areas in Europe, have demonstrated the importance of leveraging existing protocols and pipelines to successfully build a biorepository during this unprecedented pandemic [29, 30]. By rapidly amending existing protocols in collaboration with those Principal Investigators and through the volunteered time of Obstetrics and Neonatology clinicians, we were able to rapidly enroll and collect high quality, wide-ranging sample types from pregnant women and their newborns. Attempts to develop biobanks from other devastating pandemics, such as the Ebola crisis in West Africa [31], have also shown that identifying infrastructure gaps and points of cultural sensitivity are critical to achieving a successful biobank relevant to the population it serves. Data from our group and others have demonstrated that COVID-19 disease has disproportionately affected people of color and lower socioeconomic status [22, 32–35], widening health disparities. The recruitment of study staff members fluent in Spanish to facilitate the full understanding and inclusion of women from the hardest-hit communities in Massachusetts, and making changes to our enrollment and sample collection processes that sensitively responded to the stressors experienced by our pregnant and delivering women, were critical to the success and inclusivity of this study.

## Conclusions

The COVID-19 mother-newborn dyad biorepository described here will serve as an invaluable resource for further research into the impact of COVID-19 on the mother and the developing fetus. The specimens collected during the peak of the pandemic from the mother-newborn dyad and the accompanying clinical annotation have the potential to dramatically impact biological understanding of maternal inflammation and immune activation, how such processes may lead to severe maternal morbidity, and mechanisms of feto-placental vulnerability to inflammation or infection as well as mechanisms of antibody-mediated protection. To date, our understanding of the biological consequences of COVID-19 in pregnancy and clinical counseling in this regard has been largely guided by data from case reports and large case series [14–20, 36]. Biological data including rigorous and relevant control populations is urgently needed. Establishing a successful mother-newborn biorepository during the peak of the COVID-19 pandemic in Massachusetts required patient advocacy, multidisciplinary action, and creative solutions to unique challenges.

## Supplementary information

**Additional file 1. Supplementary Tables S1-S3. Table S1.** List of supplies with catalog numbers for maternal sample collection kit. **Table S2.** Samples collected from maternal subjects by COVID status. **Table S3.** Samples collected from newborn subjects by mother's COVID status.

**Additional file 2.** Instructions for collecting cord blood and placental biopsies.

**Additional file 3.** Instructions for best practices for COVID-19 specimen handling.

**Additional file 4.** Informational flier posted in clinical areas to advertise the COVID-19 biorepository.

**Additional file 5.** Consent form - adult protocol.

**Additional file 6.** Consent form - pediatric protocol.

**Additional file 7.** Instructions for assembly of maternal sample collection kit.

**Additional file 8.** REDCap data fields.

## Data Availability

The datasets analyzed during the current study are available from the corresponding author on reasonable request. For maternal samples, the Massachusetts General Hospital COVID-19 Biospecimens Access Committee has a formal process for receipt and consideration of sample requests (interested researchers should contact the Obstetrics Principal Investigator, Dr. Edlow, for details on initiating this process). Ultimate use of specimens will depend on specimen availability, collaboration with a Massachusetts General Hospital investigator, the lack of duplication with prior requests/projects, and a secondary-use IRB. For neonatal/pediatric sample requests, researchers should contact the pediatric Principal Investigator, Lael Yonker.

## Abbreviations

MGH: Massachusetts General Hospital
NICU: Neonatal intensive care unit
EMR: Electronic medical record
CRC: Clinical research coordinator
PPE: Personal protective equipment
RT-PCR: Real-time reverse transcription PCR
IRB: Institutional Review Board
PBMC: Peripheral blood mononuclear cell
