# Supplementary material for: Rapid establishment of a COVID-19 perinatal biorepository: early lessons from the first 100 women enrolled
Source: BMC Med Res Methodol. 2020 Aug 26;20:215. doi: 10.1186/s12874-020-01102-y (PMC7447612; doi:10.1186/s12874-020-01102-y)
Supplement: Supplementary file 1 — Additional file 1. Supplementary Tables S1-S3. Table S1. List of supplies with catalog numbers for maternal sample collection kit. Table S2. Samples collected from maternal subjects by COVID status. Table S3. Samples collected from newborn subjects by mother's COVID status. [file 12874_2020_1102_MOESM1_ESM.docx]

Table S1. List of supplies with catalog numbers for maternal sample collection kit.

| **Catalog #/Code** | **Item Description** |
| --- | --- |
| BD 762165 | PAXgene RNA tubes |
| BD 367863 | 6 ml EDTA tubes |
| BD 367841 | 2 ml EDTA tubes |
| BD 366643 | 10 ml EDTA tubes |
| BD 365967 | microtainers (SST) |
| BD 367987 | 7.5 ml SST tube |
| Fisher 23600956 (copan Diagnostic 518CS01) | Nylon Flocked Dry Swabs (nasal swab) |
| Copan Diagnostics 518C.BX (VWR 10755-194) | Minitip Flocked Dry Swab, Sterile,100 mm breakpoint from swab (oropharyngeal swab) |
| Corning 352059 (Fisher 1495911B) | 14 mL roundbottom test tube w/ snap cap |
| SPC400 | MediChoice specimen container (4 oz/120 cc) |
| Om Main Street (SB0609) | Biohazard specimen bag- ziplock with pocket |
| Elkay Plastics LABZ2024BC  (VWR 10789-520) | Lab Loc Specimen Bags – Large Size, zipper closure |
| 10790-078 | Biohazard bags |
| Staples 1013000 | Spoons for stool collection |
| Lab Loc Specimen Bags, Elkay Plastics | Clear biohazard bags |
| Busse 737 (Westnet 737) | Classic suture removal kit |
| Sigma-Aldrich, HSWNH181-100EA | 18-gauge needles |
| Owens&Minor, 0715003157 | Sterile gauze pads |
| Puritan 25-15061PF | Vaginal and rectal swabs |
| Fisher 02-682-558 | 2 ml screw-cap milk collection tubes |
| Medela # 87110S-50 | 35 mL Colostrum/Breastmilk Container \| Medela |
| Fisher 22-363-750 | Alcohol prep pads |
| Staples 479877 | 2x4 labels for patient kits |
| Staples 479880 | 1x 2 5/8 labels for patient kits |
| BD 302830 (westnet) | 20 cc syringe |
| Medline DYND36600 | Urine/stool collection unit/hat |
| HARDWOOD PRODUCTS 2950 | Scoopers for stool |
| USA Sci 90231615 | Placenta collection boxes |

Table S2. Samples collected from maternal subjects by COVID status

|  | **All (n=97)** | **COV19+ (n=35)** | **COV19- (n=62)** | ***P*** |
| --- | --- | --- | --- | --- |
| Maternal blood | 88 (91%) | 28 (80%) | 60 (97%) | 0.01 |
| Stool | 29 (30%) | 12 (34%) | 17 (27%) | 0.50 |
| Urine | 96 (99%) | 34 (97%) | 62 (100%) | 0.36 |
| Saliva | 76 (78%) | 23 (66%) | 53 (85%) | 0.04 |
| Sputum | 7 (7%) | 5 (14%) | 2 (3%) | 0.09 |
| Nasal swab | 77 (79%) | 23 (66%) | 54 (87%) | 0.02 |
| Oropharyngeal swab | 63 (65%) | 18 (51%) | 45 (73%) | 0.04 |
| Vaginal swab | 79 (81%) | 23 (66%) | 56 (90%) | 0.01 |
| Rectal swab | 81 (81%) | 25 (71%) | 56 (90%) | 0.02 |
| *Delivery specimens* | *n=90* | *n=29* | *n=61* |  |
| Placenta | 86 (96%) | 26 (90%) | 60 (98%) | 0.10 |
| Umbilical cord blood | 84 (93%) | 25 (86%) | 59 (97%) | 0.08 |
| Breastmilk | 20 (22%) | 4 (14%) | 16 (26%) | 0.12 |

COV19+= positive for SARS CoV-2 on RT-PCR of nasopharyngeal swab at any time during pregnancy; COV19-= negative for SARS CoV-2 on RT-PCR of nasopharyngeal swab when tested for COVID-19 symptoms or as part of universal screening protocol. Differences between groups analyzed by Chi-square test.

Table S3. Samples collected from newborn subjects by mother’s COVID status

|  | **All (n=67)** | **COV19+ (n=24)** | **COV19- (n=43)** | ***P*** |
| --- | --- | --- | --- | --- |
| Blood | 43 (64%) | 18 (75%) | 25 (58%) | 0.19 |
| Urine | 47 (70%) | 18 (75%) | 31 (72%) | 0.78 |
| Stool | 62 (93%) | 21 (88%) | 40 (95%) | 0.66 |
| Nasopharyngeal swab | 13 (19%) | 3 (13%) | 10 (23%) | 0.35 |
| Oropharyngeal swab | 21 (31%) | 7 (33%) | 14 (33%) | >0.99 |
| Tracheal aspirate | 3 (4%) | 3 (13%) | 0 (0%) | 0.04 |

COV19+= mother tested positive for SARS CoV-2 on RT-PCR of nasopharyngeal swab at any time during pregnancy; COV19-= mother tested negative for SARS CoV-2 on RT-PCR of nasopharyngeal swab when tested for COVID-19 symptoms or as part of universal screening protocol. Differences between groups analyzed by Chi-square test.
