# Supplementary material for: Rapid establishment of a COVID-19 perinatal biorepository: early lessons from the first 100 women enrolled
Source: BMC Med Res Methodol. 2020 Aug 26;20:215. doi: 10.1186/s12874-020-01102-y (PMC7447612; doi:10.1186/s12874-020-01102-y)
Supplement: Supplementary file 2 — Additional file 2. Instructions for collecting cord blood and placental biopsies. [file 12874_2020_1102_MOESM2_ESM.pdf]

COVID-19 Research Study

HOW TO COLLECT CORD BLOOD AND PLACENTAL BIOPSIES

BEFORE DELIVERY, GATHER MATERIALS:
[] 1 collection baggie and 1 collection box (located in large biohazard bags under COVID fridge in dirty utility)
[] Non-sterile gloves
[] A surface to work on – can use area near sink in L&D room or a silver cart in OR (cover with chux pad)

PART 1. Cord Blood Collection:

1. Draw 20 ccs of blood **directly off cord prior to placental detachment but after clinical collection** in a 20 cc syringe attached to 18 g needle.
2. Fill blood tubes (by puncturing tops with needle) in the following order:
    1. SST ("tiger-top" tube) with 5 mL
    2. 1st EDTA ("purple-top" tube) with 5 mL
    3. 2nd EDTA ("purple-top" tube) with 5 mL
    4. PAXgene (liquid-filled red-top tube) with 2.5 mL

    **shake tube vigorously - contents may solidify*

If you have extra blood, fill extra purple-top tubes – can obtain from L&D

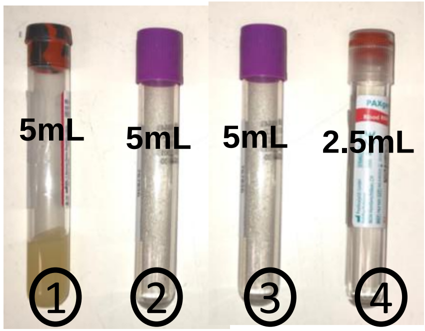

3. Using clean gloves, place tubes into 1st blue biohazard sealed bag.

PART 2: Fetal Side Placenta Biopsies:

1. Locate area central on placenta at least 3-5 cm from the cord insertion (avoid edge, calcifications, hemorrhage, masses, infarctions, vessels)
2. Use forceps and scissors to cut TWO ~1cm biopsies of placenta
3. Cut off and DISCARD membranes
4. Thoroughly rinse biopsies in petri dishes with "DPBS" (large tubes)
5. Place each piece into separate 15mL tubes labeled "Plac-F" (each box includes 2 "Plac-F" tubes, one for each biopsy)

*Ensure that biopsies are *completely submerged* in liquid

Step 1

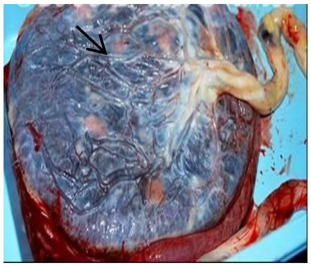

Step 2

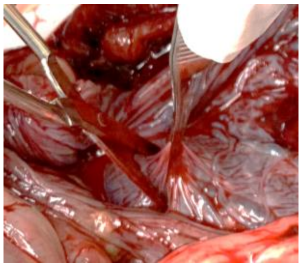

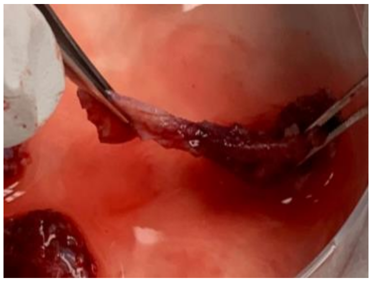

Step 3

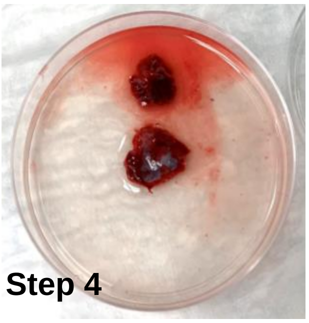

Step 4

PART 3. Maternal Side Placenta Biopsies:

1. Flip placenta over to Maternal Side
2. Blot surface with gauze to remove excess blood
3. Repeat Steps 2-5 above (for Step 3, cut off decidua which is the grayish top layer of maternal side)
4. Place each piece into separate 15mL tubes labeled "Plac-M" (each box includes 2 "Plac-M" tubes, one for each biopsy)

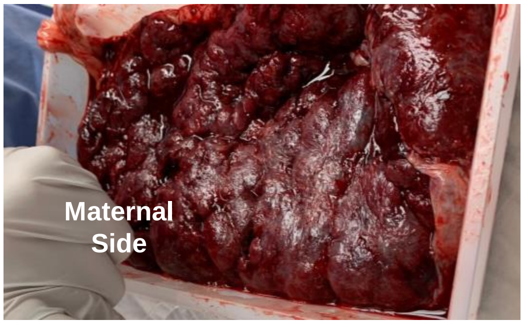

AFTER COLLECTION:
-Place 15-mL "Plac-F" and "Plac-M" tubes back inside the **white box**. Using clean gloves, place white box into labeled medium clear biohazard bag. Store box in **COVID STUDY fridge at 4°C until pickup.**
-Using clean gloves, place 1st blue biohazard sealed bag with cord blood tubes into 2nd blue biohazard sealed bag, keeping outside of 2nd bag as "clean" as possible. Store bag in **pink COVID STUDY basin on counter in dirty utility room at room temperature until pickup.**
-Complete **pathology requisition** order in Epic. Specify "**FOR COVID19 BIOREPOSITORY**" and any separate clinical indications for the pathology evaluation, and write "biopsies were taken from the placenta"
