# Supplementary material for: Rapid establishment of a COVID-19 perinatal biorepository: early lessons from the first 100 women enrolled
Source: BMC Med Res Methodol. 2020 Aug 26;20:215. doi: 10.1186/s12874-020-01102-y (PMC7447612; doi:10.1186/s12874-020-01102-y)
Supplement: Supplementary file 3 — Additional file 3. Instructions for best practices for COVID-19 specimen handling. [file 12874_2020_1102_MOESM3_ESM.pdf]

COVID-19 Perinatal Research Study

Cohort: COVID-19-Affected (Positive, At-Risk) Pregnant Individuals

BEST PRACTICES FOR COVID-19 SPECIMEN HANDLING

1. Clinician collects specimens, labels them, and places them into one of the two clean emesis basins.
2. Clinician then removes gloves, sanitizes hands, dons new gloves, and places specimens into clean biohazard bags.
3. These biohazard bags are then decontaminated with viricidal wipes prior to being placed into the second clean emesis basin for transport out of the room.
4. Once out of the room, these bags are immediately dropped into another clean biohazard bag being held by study staff and sealed.
5. This bag will then be placed into a plastic transport vessel, which will then be placed into an outer receptacle (cooler) before transport.
6. Once at the research lab, the receptacle will be handed-off to lab staff who will unpack per assigned biosafety level.
7. The emptied transport vessel and outer receptacle will be cleaned with viricidal wipes before returning to circulation. All absorbent packing material discarded.

https://www.youtube.com/watch?v=tSASmeAYZs4&feature=youtu.be
