# Supplementary material for: Rapid establishment of a COVID-19 perinatal biorepository: early lessons from the first 100 women enrolled
Source: BMC Med Res Methodol. 2020 Aug 26;20:215. doi: 10.1186/s12874-020-01102-y (PMC7447612; doi:10.1186/s12874-020-01102-y)
Supplement: Supplementary file 4 — Additional file 4. Informational flier posted in clinical areas to advertise the COVID-19 biorepository. [file 12874_2020_1102_MOESM4_ESM.pdf]

COVID-19 RESEARCH STUDY

Recruiting COVID-19-Affected (Positive, At-Risk) Pregnant Individuals

Dr. Xu Yu, Dr. Jonathan Li, Dr. Andrea Edlow
Clinical Research Coordinator: Sammy Devane
IRB Protocol #: 2020P000804

The MGH has launched a COVID-19 biorepository of various patient samples. With your help, we would like to expand this research to the pregnant population!

Who can participate?

- ALL pregnant individuals who are COVID-19-positive/at risk

How can you help?

- The goal is to collect a variety of samples from COVID-19-positive OB patients at three time points during their pregnancy/postpartum period, including at delivery.
    - Samples include blood, stool, urine, saliva, sputum, fluid, swabs of the nose, throat, vagina and/or rectum, placenta, umbilical cord blood, and/or breast milk.
    - All samples should be placed in COVID-19 study fridges in utility rooms on Blake 14, Blake 13, or Ellison 13 at the time of collection, except for **blood tubes** which should be placed into the pink study basin
    - Please page Andrea Edlow (21813) if a sample is collected postpartum

**Participating OB patients can choose to opt in or out of any and all samples*

Questions, concerns, ideas?

Please email aedlow@mgh.harvard.edu

Thank you for making this important study possible!! We could not do it without you!

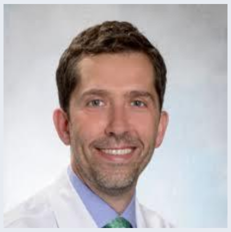

Paul Lerou, MD

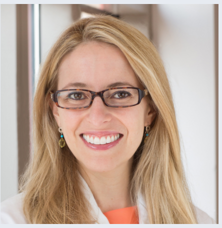

Andrea Edlow, MD

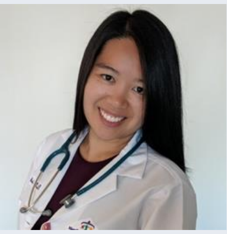

Jessica Shui, MD

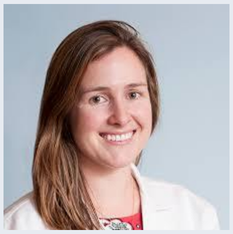

Lael Yonker, MD

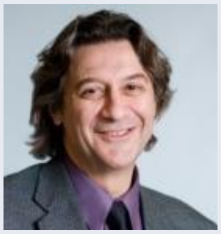

Alessio Fasano, MD

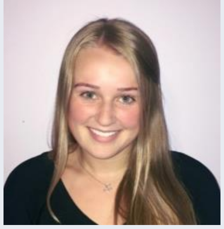

Sammy Devane, CRC

MASSACHUSETTS GENERAL HOSPITAL
