# Supplementary material for: Rapid establishment of a COVID-19 perinatal biorepository: early lessons from the first 100 women enrolled
Source: BMC Med Res Methodol. 2020 Aug 26;20:215. doi: 10.1186/s12874-020-01102-y (PMC7447612; doi:10.1186/s12874-020-01102-y)
Supplement: Supplementary file 5 — Additional file 5. Consent form - adult protocol. [file 12874_2020_1102_MOESM5_ESM.pdf]

Partners HealthCare System
Research Consent Form

Subject Identification

General Consent Form Template
Version Date: January 2019

Protocol Title: Clinical Assessment and Sampling of Individuals with or at Risk for Coronavirus Disease 2019 (COVID-19)

Principal Investigator: Xu Yu, M.D. (MGH)

Site Principal Investigator: Jonathan Li, M.D. (BWH)

Description of Subject Population: Non-Hospitalized – Minimal Risk/Verbal Consent (without BB)

About this consent form

Please read this form carefully. It tells you important information about a research study. A member of our research team will also talk to you about taking part in this research study. People who agree to take part in research studies are called "subjects." This term will be used throughout this consent form.

Partners HealthCare System is made up of Partners hospitals, health care providers, and researchers. In the rest of this consent form, we refer to the Partners system simply as "Partners."

If you decide to take part in this research study, you must sign this form to show that you want to take part. We will give you a signed copy of this form to keep.

Some of the people who are eligible to take part in this study may not be able to give consent to take part because of their medical condition. Instead we will ask the person's authorized representative to give consent. Throughout the consent form, "you" always refers to the person who takes part in the study.

Key Information

Taking part in this research study is up to you. You can decide not to take part. If you decide to take part now, you can change your mind and drop out later. Your decision won't change the medical care you get within Partners now or in the future.

The following key information is to help you decide whether or not to take part in this research study. We have included more details about the research in the Detailed Information section that follows the key information.

Subject Identification

Why is this research study being done?

The purpose of this study is to learn more about the new coronavirus (COVID-19), including its spread and its effects on the immune system. The information learnt from this study might inform the design for effective treatment or vaccine against COVID-19.

You are being asked to take part in this study because you are (1) at risk of contracting the novel coronavirus; (2) infected or presumptively infected with coronavirus, or (3) recovered from COVID-19.

How long will you take part in this research study?

The amount of time that is spent in this study may vary between a single visit (which may take approximately 0.5-2 hours) or multiple visits over 2 years depending on whether or not you are infected with COVID-19. You may withdraw at any time.

What will happen if you take part in this research study?

If you decide to join this research study, we may ask you about your medical history, and we may collect various samples, such as blood, respiratory, urine or other samples. If available, discarded samples that are collected for your clinical care may be added to the collection. If you are a household member or community participant, you may be given a kit to collect your own samples, including your own blood by a fingerstick.

Why might you choose to take part in this study?

You will not benefit from taking part in this research study. However, your participation may provide valuable information about the prevention, transmission, clinical outcomes, and treatment of COVID-19 and other diseases.

Why might you choose NOT to take part in this study?

Taking part in this research study has some risks and requirements that you should consider carefully.

Important risks and possible discomforts to know about include temporary pain from having a blood draw or weakness during the procedure.

Subject Identification

A detailed description of side effects, risks, and possible discomforts can be found later in this consent form in the section called "What are the risks and possible discomforts from being in this research study?"

If you have questions or concerns about this research study, whom can you call?

You can call us with your questions or concerns. Our telephone numbers are listed below. Ask questions as often as you want.

Drs. Xu Yu, M.D., and Jonathan Li, M.D., are the persons in charge of this study. You can call Dr. Yu at 617-879-8196 or Dr. Li at 617-407-4549 M-F from 9am-5pm. If you have questions about the scheduling of appointments or study visits, call the study coordinator team at 855-724-0557 M-F from 9am-5pm.

If you want to speak with someone **not** directly involved in this research study, please contact the Partners Human Research Committee office. You can call them at 857-282-1900.

You can talk to them about:

- Your rights as a research subject
- Your concerns about the research
- A complaint about the research
- Any pressure to take part in, or to continue in the research study

Detailed Information

Why is this research study being done?

The purpose of this study is to try to understand the spread of COVID-19 and its effects on your immune system. Understanding how the body handles the virus may lead to new approaches to treatment and vaccines.

Who will take part in this research?

We are asking you to participate in this study because you may be at risk or in different stages of the course of COVID-19 infection or suspected infection, including (1) mild illness, (2) moderate to severe illness, and (3) convalescence.

What will happen in this research study?

We may review your medical record and ask you questions about your symptoms and medical history. We may ask you to complete questionnaires about your health. We may contact you in the future to get additional information and ask if you are interested in joining other research studies.

For healthcare workers, a member of the study team will collect blood no more than monthly.

For household members and community participants, you will be asked to collect fingerstick blood samples and throat swabs, potentially at multiple time points over a 4 week period.

For participants with active disease, research samples such as blood, body tissue/fluids (such as urine, saliva, stools) and/or mucosal samples (such as nasopharyngeal and oropharyngeal swabs) may be collected daily until recovery.

For participants who are pregnant, additional research samples such as breastmilk, placental biopsies, and umbilical cord blood may be collected during your delivery admission and postpartum period. If amniotic fluid or chorionic villi are collected during your prenatal care for clinical reasons, the excess material could be used for the study and this poses no additional risk to the pregnancy or fetus. During the delivery hospitalization, placental biopsies and umbilical cord blood collection will occur after delivery and pose no risk to the pregnancy or fetus.

For participants who have recovered from the disease, research samples may be collected monthly for 1 year and every three months for 2 years.

For all participants, no more than 550 mL (approximately 2 cups) will be drawn per 8 week period. For participants in active disease, no more than 100 mL (less than half a cup) will be drawn per week. For pregnant participants, no more than 50 mL (about 3 tablespoons) will be drawn per 8 week period.

Also, as part of your routine care, your doctor may obtain blood, body tissue/fluids (including bronchoalveolar lavage) and mucosal swabs from you for testing. After the tests for your medical care are completed, part of your samples may be left over. Normally these leftover samples would be thrown away. We are asking you to allow us to collect these samples.

You may decline having any of these samples taken at any point during the study.

Study staff will assign a code number to your samples and health information. Your name, medical record number, or other information that easily identifies you will not be stored with your samples or health information. The key to the code will be stored securely in a separate file.

For what type of research will my samples be used?

This research may include laboratory tests to study COVID-19 and other infections in your blood that affect the immune system. In addition, your DNA will be extracted and studied for individual human genetic markers. DNA is inherited from both parents and it is also called the "molecule of heredity". It is a nucleic acid that contains the genetic instructions responsible for most of your inherited traits (ranging from hair color to disease susceptibility).

Genetic research may include looking at some or all of your genes and DNA to see if there are links to different types of health conditions, including COVID-19. We may ask other laboratories outside of Partners to do these tests for us. All blood and information will be coded with a number and your name will not be known. The link to the code will not be known.

Coded samples and data may be sent to other researchers who are also studying COVID-19. These researchers include but are not limited to the National Institutes of Health, Harvard, MIT, and the Broad Institute. We will keep these coded samples indefinitely or until the samples are used up.

You can change your mind at any time about allowing your samples or information to be used for future research. If you do, contact Dr. Xu Yu at xyu@mgh.harvard.edu or 617-879-8196 to let us know. Then your samples will be destroyed and no longer used for future research.

Review of Medical Records from Hospital Admissions or Emergency Department Visits
Partners has an electronic system that lets your study doctors know if you are admitted to a Partners Hospital, or if you visit a Partners Hospital Emergency Department. We want to make sure that the study doctors know about any possible problems or side effects that you experience while you are taking part in the study.

Study Information Included in Your Electronic Medical Record
A notation that you are taking part in this research study may be made in your electronic medical record. Information from the research that relates to your general medical care may be included in the record (for example, list of allergies, results of standard blood tests done at the hospital labs).

Genetic Testing
We may also perform a "whole genome analysis" on your DNA sample. Usually researchers study just a few areas of your genetic code that are linked to a disease or condition. In whole genome analyses, all or most of your genes are looked at and used by researchers to study links to immunology and infectious disease.

In order to allow researchers to share test results, the National Institutes of Health (NIH) and other central repositories have developed special data (information) banks that analyze data and collect the results of whole genome studies. These banks may also analyze and store DNA samples, as well. These central banks will store your genetic information and samples and give them to other researchers to do more studies. We do not think that there will be further risks to your privacy and confidentiality by sharing your samples and whole genome information with these banks. However, we cannot predict how genetic information will be used in the future. The samples and data will be sent with only your code number attached. Your name or other directly identifiable information will not be given to central banks. There are many safeguards in place to protect your information and samples while they are stored in repositories and used for research.

Research using your samples and whole genome information is important for the study of virtually all human diseases and conditions. Therefore, the sample/data banks will provide study data for researchers working on any disease.

How may we use and share your samples and health information for other research?

The information we collect in this study may help advance other research. Your coded samples and health information may be shared with researchers at Partners institutions. They may also be shared with researchers at non-Partners institutions or with for-profit companies that are working with Partners researchers. Your samples will not be sold for profit. You will not be asked to provide additional informed consent for these uses.

We will only share information that identifies you with researchers within Partners who have approval of the Partners ethics board. We will not share information that identifies you with researchers outside Partners. If we share your samples and/or health information with other researchers outside of Partners, we will label the samples and information with a code instead of your name or other directly identifying information.

Will you get the results of this research study?

You and your doctor should not expect to get information about the results of the research study or the results of your individual participation in the research study. We will study samples and information from many people. It could take many years before anyone knows whether the results have any meaning. There is a small chance that we could find out something from the study that might be important to your health. If this happens, we may contact you to find out if you would like to learn more. However, even if we find something important to your health, we cannot guarantee that you will be contacted.

What are the risks and possible discomforts from being in this research study?

Blood Draw: You may have a bruise (a black and blue mark) or pain where we take the blood samples. There is also a small risk of feeling lightheaded, fainting or infection.

Repeated blood donation can lead to iron deficiency. This can cause an individual to feel weak and/or tired and may require taking an iron supplement. The timing and frequency of blood collection as well as the quantity of blood collected will be recorded and closely monitored to avoid these problems.

Fingerstick blood collection: Fingersticks may cause a small amount of pain. Rarely, people faint during or after seeing blood. The amount of blood taken is very small.

Sputum or other respiratory sample collection: Sputum or other respiratory sample collection may cause mild discomfort or coughing. Bronchoalveolar lavage and other fluid may be collected if deemed appropriate by your clinical team.

Swabs: Getting swabs of your mouth, nose, vagina and/or rectum may result in slight irritation of the sampling sites. Collecting swabs from your nose may cause mild discomfort, watery eyes, or sneezing. Additionally, you may experience a dry area in the mouth at the sampling site for a short period of time (less than 5 minutes). Collecting swabs from the vagina or rectum may cause discomfort.

Saliva Collection: You may experience a dry area in your mouth for up to five minutes after providing a saliva sample.

Stool Samples: In providing a stool sample there is a risk of contamination of skin with feces from the collection container.
For Pregnant or Postpartum Women: The effect of blood draws less than 50 ml in an 8-week period and swab collections have no known risks to the woman or the fetus. Cord blood, amniotic fluid/chorionic villi, placenta, and other specimens that are collected during clinical care and would otherwise be discarded, pose no risk to the pregnancy or the fetus. Collecting breastmilk may cause mild discomfort if hand expression or breastpumping is uncomfortable for you. Discomfort is usually minimal. Collection of breastmilk will depend on mother's supply and will only be performed if it will not interfere with the baby's breastmilk needs.

Anxiety: Subjects may experience some concern or anxiety regarding maintaining confidentiality of study information.

Privacy: A risk of allowing us to use your samples and health information for research is a potential loss of privacy. We protect your privacy by coding your samples and health information. To minimize the risks associated with genetic testing, no results obtained in this study will not be placed in your medical record and no research results will be given to you or your healthcare providers.

What are the possible benefits from being in this research study?

There are no direct benefits to you from participating in this research study. We hope that others may benefit in the future if we can learn more about the immune responses to COVID-19.

Can you still get medical care within Partners if you don't take part in this research study, or if you stop taking part?

Yes. Your decision won't change the medical care you get within Partners now or in the future. There will be no penalty, and you won't lose any benefits you receive now or have a right to receive.

We will tell you if we learn new information that could make you change your mind about taking part in this research study.

What should you do if you want to stop taking part in the study?

If you take part in this research study, and want to drop out, you should tell us. We will make sure that you stop the study safely. We will also talk to you about follow-up care, if needed.

Also, it is possible that we will have to ask you to drop out of the study before you finish it. If this happens, we will tell you why. We will also help arrange other care for you, if needed.

Will you be paid to take part in this research study?

You will not receive payment for your participation. If you drive to the study visit, you may receive a parking sticker to cover the length of your visit.

We may use your samples and information to develop a new product or medical test to be sold. The Sponsor, hospital, and researchers may benefit if this happens. There are no plans to pay you if your samples or information are used for this purpose.

What will you have to pay for if you take part in this research study?

Study funds will pay for certain study-related items and services. We may bill your health insurer for, among other things, routine items and services you would have received even if you did not take part in the research. You will be responsible for payment of any deductibles and co-payments required by your insurer for this routine care or other billed care. If you have any questions about costs to you that may result from taking part in the research, please speak with the study doctors and study staff. If necessary, we will arrange for you to speak with someone in Patient Financial Services about these costs.

What happens if you are injured as a result of taking part in this research study?

We will offer you the care needed to treat any injury that directly results from taking part in this research study. We reserve the right to bill your insurance company or other third parties, if appropriate, for the care you get for the injury. We will try to have these costs paid for, but you may be responsible for some of them. For example, if the care is billed to your insurer, you will be responsible for payment of any deductibles and co-payments required by your insurer.

Injuries sometimes happen in research even when no one is at fault. There are no plans to pay you or give you other compensation for an injury, should one occur. However, you are not giving up any of your legal rights by signing this form.

If you think you have been injured or have experienced a medical problem as a result of taking part in this research study, tell the person in charge of this study as soon as possible. The researcher's name and phone number are listed in the beginning of this consent form.

If you take part in this research study, how will we protect your privacy?

Federal law requires Partners to protect the privacy of health information and related information that identifies you. We refer to this information as “identifiable information.”

In this study, we may collect identifiable information about you from:

- Past, present, and future medical records
- Research procedures, including research office visits, tests, interviews, and questionnaires

Subject Identification

Who may see, use, and share your identifiable information and why they may need to do so:

- Partners researchers and staff involved in this study
- The sponsor(s) of the study, and people or groups it hires to help perform this research or to audit the research
- Other researchers and medical centers that are part of this study
- The Partners ethics board or an ethics board outside Partners that oversees the research
- A group that oversees the data (study information) and safety of this study
- Non-research staff within Partners who need identifiable information to do their jobs, such as for treatment, payment (billing), or hospital operations (such as assessing the quality of care or research)
- People or groups that we hire to do certain work for us, such as data storage companies, accreditors, insurers, and lawyers
- Federal agencies (such as the U.S. Department of Health and Human Services (DHHS) and agencies within DHHS like the Food and Drug Administration, the National Institutes of Health, and the Office for Human Research Protections), state agencies, and foreign government bodies that oversee, evaluate, and audit research, which may include inspection of your records
- Public health and safety authorities, if we learn information that could mean harm to you or others (such as to make required reports about communicable diseases or about child or elder abuse)
- Other:

Some people or groups who get your identifiable information might not have to follow the same privacy rules that we follow and might use or share your identifiable information without your permission in ways that are not described in this form. For example, we understand that the sponsor of this study may use your identifiable information to perform additional research on various products or conditions, to obtain regulatory approval of its products, to propose new products, and to oversee and improve its products' performance. We share your identifiable information only when we must, and we ask anyone who receives it from us to take measures to protect your privacy. The sponsor has agreed that it will not contact you without your permission and will not use or share your identifiable information for any mailing or marketing list. However, once your identifiable information is shared outside Partners, we cannot control all the ways that others use or share it and cannot promise that it will remain private.

Because research is an ongoing process, we cannot give you an exact date when we will either destroy or stop using or sharing your identifiable information. Your permission to use and share your identifiable information does not expire.

The results of this research study may be published in a medical book or journal, or used to teach others. However, your name or other identifiable information **will not** be used for these purposes without your specific permission.

Your Privacy Rights

You have the right **not** to sign this form that allows us to use and share your identifiable information for research; however, if you don't sign it, you can't take part in this research study.

You have the right to withdraw your permission for us to use or share your identifiable information for this research study. If you want to withdraw your permission, you must notify the person in charge of this research study in writing. Once permission is withdrawn, you cannot continue to take part in the study.

If you withdraw your permission, we will not be able to take back information that has already been used or shared with others, and such information may continue to be used for certain purposes, such as to comply with the law or maintain the reliability of the study.

You have the right to see and get a copy of your identifiable information that is used or shared for treatment or for payment. To ask for this information, please contact the person in charge of this research study. You may only get such information after the research is finished.

Informed Consent and Authorization

Statement of Person Giving Informed Consent and Authorization

- I have read this consent form.
- This research study has been explained to me, including risks and possible benefits (if any), other possible treatments or procedures, and other important things about the study.
- I have had the opportunity to ask questions.
- I understand the information given to me.

Subject Identification
