# Supplementary material for: Rapid establishment of a COVID-19 perinatal biorepository: early lessons from the first 100 women enrolled
Source: BMC Med Res Methodol. 2020 Aug 26;20:215. doi: 10.1186/s12874-020-01102-y (PMC7447612; doi:10.1186/s12874-020-01102-y)
Supplement: Supplementary file 6 — Additional file 6. Consent form - pediatric protocol. [file 12874_2020_1102_MOESM6_ESM.pdf]

Subject Identification

Protocol Title: Specimen Collection in Patients with COVID-19, and Healthy Controls

Principal Investigator: Lael Yonker MD

Site Principal Investigator:

Description of Subject Population: Patients with or suspected to have COVID-19, and Healthy Controls

Collection of Samples and Health Information for Research

About this consent form

Please read this form carefully. It tells you important information about the collection and storage of tissue samples for research. A member of our research team will also talk to you about taking part in this research study. People who agree to take part in research studies are called "subjects." This term will be used throughout this consent form.

Partners HealthCare System is made up of Partners hospitals, health care providers, and researchers. In the rest of this consent form, we refer to the Partners system simply as "Partners."

If you decide to take part in this research study, you must sign this form to give your permission. We will give you a signed copy of this form to keep.

Some of the people who are eligible to take part in this study may not be able to give consent because they are less than 18 years of age (minors). Instead we will ask the parent or guardian of the child to give consent. Throughout the consent form, "you" always refers to the person who takes part in the study.

Key Information

Taking part in this research study is up to you. You can decide not to take part. If you decide to take part now, you can change your mind and drop out later. Your decision won't change the medical care you get within Partners now or in the future.

Subject Identification

The following key information is to help you decide whether or not to take part in this research study. We have included more details about the research in the Detailed Information section that follows the key information.

What is the purpose of this research tissue bank?

The purpose of this research tissue bank is to collect, process, and store samples until researchers need them to do research. Tissue samples in this bank will be used mainly for COVID-19 research. Research tissue banks collect and store many types of samples, such as blood, urine or other bodily material.

Our research tissue bank is located at 55 Fruit St., Jackson 14, Boston MA 02114. There is no set limit to the number of individuals who provide samples to this bank. The more samples and health information that we can collect, the more useful the tissue bank will be for research.

What will happen if you take part in this research study?

If you decide to join this research study, you will indicate what specimen we can collect from you later in the form.

Why might you choose to take part in this research tissue bank?

You will not directly benefit from research conducted on your samples stored in the research tissue bank. We hope that research using the samples and information will help us understand, prevent, treat, or cure the illnesses and conditions studied.

Why might you choose NOT to take part in this research tissue bank?

Taking part in this research tissue bank has some risks that you should consider carefully.

The main risk of allowing us to store and use your samples and certain limited health information for research is a potential loss of privacy. A detailed description of the risks can be found later in this consent form in the section called “What are the risks and possible discomforts from being in this research study?”.

If you have questions or concerns about this research tissue bank, whom can you call?

You can call us with your questions or concerns. Our telephone numbers are listed below. Ask questions as often as you want.

Lael Yonker, MD is the person in charge of this the research tissue bank. You can call her at (617) 726-3719, M-F 9-5, with questions about this research study.

If you want to speak with someone **not** directly involved in this research study, please contact the Partners Human Research Committee office. You can call them at 857-282-1900.

You can talk to them about:

- Your rights as a research subject
- Your concerns about the research
- A complaint about the research
- Any pressure to take part in, or to continue in the research study

Detailed Information

What will happen if you take part in this research tissue bank?

As part of your routine care, your doctor will obtain different specimens from you for testing, including blood, urine, stool, sputum (phlegm), nasal polyps, lung fluid or lung tissue samples. After the tests for your medical care are completed, part of your samples may be left over. Normally these leftover samples would be thrown away. We are asking you to allow us to collect and store this leftover blood, sputum, or tissue samples in a research tissue bank.

We may ask you to provide a sample which is not part of your routine care. We will always try to collect of research samples at the same time your doctor is doing tests for your medical care.

If you agree, the samples will be frozen and sent to the bank. We are also asking for your permission to store some of your health information with your samples so that your samples will be more useful for research. We plan to continue to review your medical record to update your health information in the tissue bank computer database.

We would like to obtain the following specimens from you today. If you are hospitalized, we would like to collect samples every day during your admission. Please initial next to each one that you will let us take.

The following specimens we would like to collect could be genotyped in some research instances. To be "genotyped" means to read the DNA of the cells in the sample. We plan to do genetic research on the DNA in your tissue sample. DNA is the material that makes up your genes. All living things are made of cells. Genes are the part of cells that contain the instructions which tell our bodies how to grow and work, and determine physical characteristics such as hair and eye color. Genes are passed from parent to child.

Blood: We would like to collect and study and/or store blood. ☐ **Not applicable**

☐ Collection of blood for research purposes **done at same time as clinical labs** (heel stick for newborns). No extra blood draws.
Subject initials____

☐ Collection of blood **for research purposes** (will involve a needle poke).
Subject initials____

☐ No collection of blood. **Subject initials____**

The following guidelines will be used for blood drawn (in an 8-week period):

- 0-6 months old 5 ml (about 1 teaspoon)
- 6-12 months old 10 ml (about 2 teaspoons)
- 12-24 months old 20 ml (about 4 teaspoons)
- Older than 2 years no more than 3 tablespoons based on your age and weight

We will not exceed 50 ml (about 10 teaspoons) in an 8-week period.

Nasopharyngeal Swab: We would like to collect a nasopharyngeal swab for research purposes. We will use a swab (Q-tip) touch the back of your throat by going through your nose. A specimen which is needed for culture for your care will be collected before research specimen**.**

☐ **Yes** ☐ **No** ☐ **Not applicable** **Subject initials_____**

Nasopharyngeal Suction: If nasopharyngeal suctions are performed, we would like to collect the secretions for research purposes. Nasopharyngeal suction is part of standard newborn care to clear secretions from the nose and will not be done for research purposes only.

☐ **Yes** ☐ **No** ☐ **Not applicable** **Subject initials_____**

Throat Swab: We would like to collect a throat swab for research purposes. You will be asked to cough several times and then we will touch the back of your throat with a swab (Q-tip). Any specimen which is needed for culture for your care will be collected before the research specimen.

☐ **Yes** ☐ **No** ☐ **Not applicable** **Subject initials_____**

Sputum: We would like to collect and study and/or store a sputum specimen for research purposes. Any specimen which is needed for culture for your care will be collected before the research specimen. We will only collect a sputum sample if you can provide one spontaneously.

☐ **Yes** ☐ **No** ☐ **Not applicable** **Subject initials_____**

Urine: We would like to collect and study and/or store a urine specimen for research purposes. Any specimen which is needed for clinical care will be collected before the research specimen.

☐ **Yes** ☐ **No** ☐ **Not applicable** **Subject initials_____**

Stool: We would like to collect and study and/or store a stool specimen for research purposes. Any specimen which is needed for clinical care will be collected before the research specimen.

☐ **Yes** ☐ **No** ☐ **Not applicable** **Subject initials_____**

Other specimens: We would like to collect and study and/or store cord blood/ placenta pathology for research purposes. Any specimen which is needed for clinical care will be collected before the research specimen.

☐ **Yes** ☐ **No** ☐ **Not applicable** **Subject initials_____**

Lung fluid. If you are having a bronchoscopy, we would like to collect fluid from your lungs (Bronchoalveolar lavage (BAL)). We will only obtain this fluid if you are having a bronchoscopy for clinical purposes and have left over BAL fluid that is collected but is not needed for clinical tests. If you are intubated during this hospitalization, or have a tracheostomy in place, we would like to collect fluid that is obtained through tracheal washings. Endotracheal tubes and tracheostomys are typically suctioned through the day as routine care. We will only obtain this fluid if you are having a suctioning done for clinical purposes and have left over fluid.

☐ **Yes** ☐ **No** ☐ **Not applicable** **Subject initials_____**

Bacteria: You will be having a culture of a sputum or lung fluid specimen. We would like to study and/or store the bacteria which grow from the specimen.

☐ **Yes** ☐ **No** ☐ **Not applicable** **Subject initials_____**

If you are hospitalized, we would like to collect your specimens on a daily basis until discharge. You have the right to decline specimen collect at any time.

We would also like your permission for the tissue bank staff to review your medical records and to contact you in the future for more information about your medical condition or health status. This could be at a medical visit or by phone to get updated information.

We may like to re-collect your specimens at future visits. If so, the research coordinator or physician may contact you to ask if you are willing to give more samples. You may decide whether or not to provide another research sample at that time. If interested, you will be asked to sign this consent form again.

We may also ask you some questions about different risk factors associated with COVID. The questionnaire asks questions about any symptoms you have had, your living situation, history of

smoking and other questions about exposure to COVID. This questionnaire will take 5-8 minutes to answer and your answers will only be seen by the staff involved in the study.

How are your samples stored?

Staff at the bank will assign your sample a code number and store it in a freezer. They will not keep your name or other information that could identify you with your sample. They will use the code number to connect your sample to your health information that is stored in a computer database. The computer database is protected with a password. Only staff at the bank will know the password.

Which researchers can use your samples and what information about you can they have?

Your samples will be made available to researchers at MGH (Massachusetts General Hospital), and other Partners institutions, as well as non-Partners academic institutions. Occasionally, your samples may be shared with for-profit companies that are working with MGH, or other Partners researchers on a specific research project. Your samples will not be sold to anyone for profit. The tissue bank will usually provide samples with limited information that does not directly identify you.

- As described above, all of the samples stored in the bank are labeled with a code number that connects the sample to medical information related to the sample. The key to the code that links the samples and information to a specific individual will only be available to the tissue bank staff, and will be securely stored.

- Researchers at Partners institutions, whose studies have been approved by the hospital ethics board, may be allowed to review your medical record to collect more health information about you. The ethics board is a group that independently reviews and watches over all research studies involving people. The board follows state and federal laws and codes of ethics to make sure that the rights and welfare of people taking part in research studies are protected.

- Researchers outside of MGH and BWH will not be given the key to the code that links your sample and medical information to your name or other direct identifiers.

For what type of research will your samples be used?

Your samples and information will be used to study COVID-19 immune responses. The long-term goals of the research are to learn how to better understand, prevent, diagnose or treat infections in the airway. It is not possible to list every research project. Also, we cannot predict all of the research questions that will be important over the next years. As we learn more, there are new research questions and new types of research may be done.

We plan to do genetic research on the DNA in your tissue sample, if you agree to allow us to take a sample of tissue that can be genotyped. DNA is the material that makes up your genes. All living things are made of cells. Genes are the part of cells that contain the instructions which tell our bodies how to grow and work, and determine physical characteristics such as hair and eye color. Genes are passed from parent to child.

In order to allow researchers to share test results, the National Institutes of Health (NIH) and other central repositories have developed special data (information) banks that analyze data and collect the results of whole genome studies. These banks may also analyze and store DNA samples, as well. These central banks will store your genetic information and samples and give them to other approved and qualified researchers to do more studies. We do not think that there will be further risks to your privacy and confidentiality by sharing your samples and whole genome information with these banks. However, we cannot predict how genetic information will be used in the future. The samples and data will be sent with only your code number attached. Your name or other directly identifiable information will not be given to central banks. There are many safeguards in place to protect your information and samples while they are stored in repositories and used for research.

Your samples and information may also be used for research on other conditions; for example, as comparisons to other diseases.

Your tissue sample may be used to create a living tissue sample (called a “cell line”) that can be grown in the laboratory. This allows researchers to have an unlimited supply of your cells in the future without asking for more samples from you.

We may use the cells taken from your blood or nasal swabs to to create a type of cell known as a pluripotent cell. This type of cell can be used to create different types of tissue, including lung cells. Your cells might be used in research involving genetic alteration of the cells. Your cells might be mixed with other human cells, mixed with animal cells, or grown in lab animals like mice.

We may also perform a whole genome analysis on your DNA sample. Usually researchers study just a few areas of your genetic code that are linked to a disease or condition. In whole genome studies, all or most of your genes are analyzed and used by researchers to study links to many diseases affected by infections in the airway.

Research using your samples and whole genome information is important for the study of virtually all diseases and conditions. Therefore, the sample/data banks will provide study data for researchers working on any disease.

How long will your samples and information be kept?

There is no scheduled date on which your samples and information in the bank will be destroyed. Your samples may be stored for research until they are "used up."

Can you stop allowing your samples and information to be stored and used for research?

Yes. You have a right to withdraw your permission at any time. If you do, your samples and your information will be destroyed. However, it will not be possible to destroy samples and information that have already been given to researchers. If you decide to withdraw your permission, you should contact the tissue bank's staff in writing at: Joey O'Donnell CF Center, CF Research Office, 275 Cambridge St., POB 5th Floor, Boston, MA 02114

If you are under 18 years at the time you consent to allow your samples to join the tissue bank, we will ask you to re-consent when you turn 18 years old and become a legal adult. If you consent now, you can decide not to continue to take part in the tissue bank when you turn 18, or at any time.

If we cannot locate you when we try to have you sign re-consent at age 18, your samples will be de-identified and will remain in the bank. If you decide not to sign re-consent, your samples and information will be withdrawn from the bank and destroyed. It will not be possible to destroy or withdraw the samples and information that have already been shared with other researchers.

Will you get results of research done using your samples?

No. The research study we are doing is only a stepping stone in understanding inflammatory responses. Therefore, no information about the results of this research study or the results of your individual participation in the research study will be given to you or your doctor. Tests

done for the research using your samples will not be useful in directing your medical treatment. The results of the tests will not be placed in your medical record.

What are the risks?

Risks of Allowing us to Store and Use Samples

The main risk of allowing us to store and use your samples and certain limited health information for research is a potential loss of privacy. We will protect your privacy by labeling your samples and information only with a code, and keeping the key to the code in a password protected database.

Information that could be used to identify you will only be shared with researchers within Partners who have approval of the Partners ethics board. Information that likely could be used to identify you will not be shared with researchers outside Partners.

Risks of Genetic Testing

Genetic information that results from this study does not have medical or treatment importance at this time. However, there is a risk that information about taking part in a genetic study may influence insurance companies and/or employers regarding your health. To further safeguard your privacy, genetic information obtained in this study will not be placed in your medical record.

Taking part in a genetic study may also have a negative impact on family or other relationships. If you do not share information about taking part in this study, you will reduce this risk.

Your doctor will explain the risks of the routine medical procedure you are having. In some cases, your doctor will ask you to sign a separate clinical consent form that explains the risks of the procedure. Allowing your samples to be placed in the bank will not change the risks of the medical procedure itself.

Risks of Blood Sample Collection

You may have a bruise (a black and blue mark) bleeding from skin puncture, or pain where we take the blood samples. There is also a small risk of infection, light-headedness, and/or fainting.

Risks of Obtaining Samples

Your doctor will explain the risks of the routine medical procedure you are having. Allowing your samples to be placed in the bank will not change the risks of the medical procedure itself.

Risks of Sputum Sample

Producing a sputum sample may cause coughing, bronchospasm and/or chest pain.

What are the costs to you to take part in the research tissue bank?

There is no cost to you to have your samples in the bank or for the research using your samples. The medical care you received that resulted in these leftover samples will be billed as usual to you and your health insurance company.

Will you be paid for your samples?

We will not pay you to allow us to store your samples and to allow research to be done with your samples.

We may use your samples and information to develop a new product or medical test to be sold. The Sponsor, hospital, and researchers may benefit if this happens. There are no plans to pay you if your samples or information are used for this purpose.

Can you still get medical care within Partners if you don't take part in this research tissue bank or if you stop taking part?

Yes. Your decision won't change the medical care you get within Partners now or in the future. There will be no penalty, and you won't lose any benefits you receive now, or have a right to receive.

Taking part in the bank is up to you. You can decide not to allow your samples and information to be placed in the bank. If you decide to take part now, you can change your mind and drop out later.

What happens if you are injured as a result of taking part in this research study?

We will offer you the care needed to treat any injury that directly results from taking part in this research study. We reserve the right to bill your insurance company or other third parties, if appropriate, for the care you get for the injury. We will try to have these costs paid for, but you may be responsible for some of them. For example, if the care is billed to your insurer, you will be responsible for payment of any deductibles and co-payments required by your insurer.

Injuries sometimes happen in research even when no one is at fault. There are no plans to pay you or give you other compensation for an injury, should one occur. However, you are not giving up any of your legal rights by signing this form.

If you think you have been injured or have experienced a medical problem as a result of taking part in this research study, tell the person in charge of this study as soon as possible. The researcher's name and phone number are listed in the next section of this consent form.

If you take part in this research study, how will we protect your privacy?

Federal law requires Partners to protect the privacy of health information and related information that identifies you. We refer to this information simply as "identifiable information."

In this study, we may collect health information about you from:

- Past, present, and future medical records
- Research procedures, including research office visits, tests, interviews, and questionnaires

Who may see, use, and share your identifiable health information and why they may need to do so:

- Partners research staff involved in this study
- The sponsor(s) of this study, and the people or groups it hires to help perform this research
- Other researchers and medical centers that are part of this study and their ethics boards
- A group that oversees the data (study information) and safety of this research
- Non-research staff within Partners who need this information to do their jobs (such as for treatment, payment (billing), or health care operations)
- The Partners ethics board that oversees the research and the Partners research quality improvement programs.
- People from organizations that provide independent accreditation and oversight of hospitals and research

- People or groups that we hire to do work for us, such as data storage companies, insurers, and lawyers
- Federal and state agencies (such as the Food and Drug Administration, the Department of Health and Human Services, the National Institutes of Health, and other US or foreign government bodies that oversee or review research)
- Public health and safety authorities (for example, if we learn information that could mean harm to you or others, we may need to report this, as required by law)
- Other:

Some people or groups who get your health information might not have to follow the same privacy rules that we follow and might use or share your health information without your permission in ways that are not described in this form. For example, we understand that the sponsor of this study may use your health information to perform additional research on various products or conditions, to obtain regulatory approval of its products, to propose new products, and to oversee and improve its products' performance. We share your health information only when we must, and we ask anyone who receives it from us to take measures to protect your privacy. The sponsor has agreed that it will not contact you without your permission and will not use or share your information for any mailing or marketing list. However, once your information is shared outside Partners, we cannot control all the ways that others use or share it and cannot promise that it will remain private.

Because research is an ongoing process, we cannot give you an exact date when we will either destroy or stop using or sharing your health information. Your permission to use and share your identifiable information does not expire.

The results of this research study may be published in a medical book or journal, or used to teach others. However, your name or other identifying information **will not** be used for these purposes without your specific permission.

Your Privacy Rights

You have the right **not** to sign this form that allows us to use and share your health information for research; however, if you don't sign it, you can't take part in this research study.

You have the right to withdraw your permission for us to use or share your health information for this research study. If you want to withdraw your permission, you must notify the person in charge of this research study in writing. Once permission is withdrawn, you cannot continue to take part in the study.

If you withdraw your permission, we will not be able to take back information that has already been used or shared with others.

You have the right to see and get a copy of your health information that is used or shared for treatment or for payment. To ask for this information, please contact the person in charge of this research study. You may only get such information after the research is finished.

Informed Consent and Authorization for Collection of Samples and Health Information for Research

Statement of Study Doctor or Person Obtaining Consent

- I have explained the research to the study subject.
- I have answered all questions about this research study to the best of my ability.

____ _______________
Study Doctor or Person Obtaining Consent Date/Time

Witness to Consent of Subjects Who Are Consenting Verbally (COVID-19 Precautions)

Statement of Witness

I represent that the consent form was presented orally to the subject in the subject's own language, that the subject was given the opportunity to ask questions, and that the subject has indicated his/her consent, assent (if applicable), and authorization for participation by (check one box as applicable):

☐ Verbal consent and assent (if applicable) over the telephone (COVID-19 precautions)

☐ Other means ____
(fill in above)

____ _______________
Witness Date/Time

Subject Identification

Witness relationship to person giving informed consent

Statement of Person Giving Informed Consent and Authorization

- I have read this consent form.
- This research study has been explained to me, including risks and possible benefits (if any), other possible treatments or procedures, and other important things about the study.
- I have had the opportunity to ask questions.
- I understand the information given to me.

Signature of Subject:

I give my consent to take part in this research study and agree to allow my health information to be used and shared as described above.

If you are consenting over the telephone, please indicate to the researcher if you agree to participate in this research.

Subject   Date/Time

Signature of Parent(s)/Guardian for Child:

I give my consent for my child to take part in this research study and agree to allow his/her health information to be used and shared as described above.

If you are consenting over the telephone, please indicate to the researcher if you agree for your child to participate in this research.

Parent(s)/Guardian for Child   Date/Time

Assent

Statement of Person Giving Assent

- This research study has been explained to me, including risks and possible benefits (if any), other possible treatments or procedures, and other important things about the study.
- I have had the opportunity to ask questions, and my questions have been answered.

Signature of Child:

I agree to take part in this research study and agree to allow my health information to be used and shared as described above.

If you are consenting over the telephone, please indicate to the researcher if you agree to participate in this research.

____ ________________
Child, Ages 14-17 Date/Time

Signature of Adult:

I agree to take part in this research study and agree to allow my health information to be used and shared as described above.

If you are consenting over the telephone, please indicate to the researcher if you agree to participate in this research.

____ ________________
Adult Date/Time

Consent of Non-English Speaking Subjects Using the "Short Form" in the Subject's Spoken Language

Statement of Hospital Medical Interpreter

Subject Identification

As someone who understands both English and the language spoken by the subject, I interpreted, in the subject's language, the researcher's presentation of the English consent form. The subject was given the opportunity to ask questions.

____ ________________

Hospital Medical Interpreter Date/Time

OR

Statement of Other Individual (Non-Interpreter)

As someone who understands both English and the language spoken by the subject, I represent that the English version of the consent form was presented orally to the subject in the subject's own language, and that the subject was given the opportunity to ask questions.

____ ________________

Name Date/Time

Consent Form Version: 2020-05-07 Biobanking ICF v1.3 (COVID-19/Healthy Controls)
