# Supplementary material for: Rapid establishment of a COVID-19 perinatal biorepository: early lessons from the first 100 women enrolled
Source: BMC Med Res Methodol. 2020 Aug 26;20:215. doi: 10.1186/s12874-020-01102-y (PMC7447612; doi:10.1186/s12874-020-01102-y)
Supplement: Supplementary file 7 — Additional file 7. Instructions for assembly of maternal sample collection kit. [file 12874_2020_1102_MOESM7_ESM.pdf]

COVID-19 Research Study

Cohort: COVID-19-Affected (Positive, PUI, At-Risk) Pregnant Individuals

HOW TO ASSEMBLE SAMPLE KITS

L&D KIT

Materials:

1. **1 large clear biohazard bag**
2. **1 blood kit, urine kit, stool kit, saliva kit, sputum kit, nasal/pharyngeal swab kit, rectal swab kit, vaginal swab kit, cord blood/placenta kit**
3. **1 medium-sized biohazard bag** [for final specimen transport after collection]
4. **1 "How to Collect Specimens" sheet**
5. **1 "Sample Collection Form"**
6. **1 "Sample Kit Labels (L&D)" label sheet and 1 "Sample Collection Container Labels" sheet**
7. **1 "Sample Kit Bag Labels" label sheet**

Steps:

1. Label 1 **large clear biohazard bag** and 1 **medium-sized clear biohazard bag** with large-font study ID label (######).
2. Unseal large clear biohazard bag and place 1 **stool collection hat** inside.
3. Place all indicated sample kits in their respective blue biohazard bags inside the large clear biohazard bag and seal closed.
4. Label "**Sample Collection Form**" with study ID label (######) next to **Study ID:** (top left of page)
5. Include "**How to Collect Specimens**," "**Sample Collection Form**," and **medium-sized clear biohazard bag** all folded in front pocket of large clear biohazard bag.

ANTEPARTUM KIT

Materials:

8. **2 medium-sized clear biohazard bags**
9. **1 blood kit, urine kit, stool kit, saliva kit, sputum kit, nasal/pharyngeal swab kit, rectal swab kit, vaginal swab kit**
10. **1 "How to Collect Specimens" sheet**
11. **1 "Sample Collection Form"**
12. **1 "Sample Kit Labels (AP)" label sheet**
13. **1 "Sample Kit Bag Labels" label sheet**
14. **1 "Sample Collection Container Labels" sheet**

Steps:

6. Label 2 **medium-sized clear biohazard bags** with large-font study ID label (######).
7. Unseal 1 medium-sized biohazard bag and place 1 **stool collection hat** inside.
8. Place all indicated sample kits in their respective blue biohazard bags inside that medium-sized clear biohazard bag and seal closed.
9. Label "**Sample Collection Form**" with study ID label (######) next to **Study ID:** (top left of page)
10. Include "**How to Collect Specimens**," "**Sample Collection Form**," and other **medium-sized clear biohazard bag** all folded in front pocket of large clear biohazard bag.

COVID-19 Research Study

Cohort: COVID-19-Affected (Positive, PUI, At-Risk) Pregnant Individuals

INDIVIDUAL SAMPLE KITS (within L&D / Antepartum Kit)

BLOOD KIT

Materials:

1. **1 blue biohazard bag**
2. **1 SST** (tiger-top tube)
3. **2 EDTA** (purple-top tube)
4. **1 PAXgene** (liquid-filled red-top tube)

Steps:

1. Label 1 **blue biohazard bag** with study ID label (######) and sample kit bag label (Blood).
2. Label 1 **SST** and 2 **EDTA** with "###### Blood – M *5 mL*" label.
3. Label 1 **PAXgene** with "###### Blood – M *2.5 mL*" label.
4. Place all **4** tubes into blue biohazard bag and seal closed.

URINE KIT

Materials:

1. **1 blue biohazard bag**
2. **1 Plastic collection container** (gray lid)

Steps:

1. Label 1 **blue biohazard bag** with study ID label (######) and sample kit bag label (Urine).
2. Label 1 **plastic collection container** with "###### Urine" label (container) and "Urine" label (lid).
3. Place container into blue biohazard bag and seal closed.

STOOL KIT

Materials:

1. **1 blue biohazard bag**
2. **1 Stool collection hat**
3. **1 Plastic collection container** (gray lid)
4. **1 Plastic spoon**

Steps:

1. Label 1 **blue biohazard bag** with study ID label (######) and sample kit bag label (Stool)
2. Label 1 **plastic collection container** with "###### Stool" label (container) and "Stool" label (lid).
3. Place container and **plastic spoon** into blue biohazard bag and seal closed.

****Stool collection hat** goes directly into large clear biohazard bag.

SALIVA KIT

Materials:

1. **1 blue biohazard bag**
2. **1 Plastic collection container** (gray lid)

Steps:

1. Label 1 **blue biohazard bag** with study ID label (######) and sample kit bag label (Saliva).
2. Label 1 **plastic collection container** with "###### Saliva" label (container) and "Saliva" label (lid).
3. Place container into blue biohazard bag and seal closed.

COVID-19 Research Study

Cohort: COVID-19-Affected (Positive, PUI, At-Risk) Pregnant Individuals

SPUTUM KIT

Materials:

1. 1 **blue biohazard bag**
2. 1 **Plastic collection container** (gray lid)

Steps:

1. Label 1 **blue biohazard bag** with study ID label (######) and sample kit bag label (Sputum).
2. Label 1 **plastic collection container** with "###### Sputum" label (container) and "Sputum" label (lid).
3. Place container into blue biohazard bag and seal closed.

NASAL/PHARYNGEAL SWAB KIT

Materials:

1. 1 **blue biohazard bag**
2. 1 **smaller swab** (nasal)
3. 1 **larger swab** (pharyngeal)
4. 2 **15-mL falcon tubes** (orange top) **with 3 mL DPBS each**
5. 2 **rubber bands**

Steps:

1. Label 1 **blue biohazard bag** with study ID label (######) and sample kit bag labels (Nasal Swab and Pharyngeal Swab).
2. Label 1 **15-mL falcon tube** (with 3 mL DPBS) with "###### Nasal Swab" label. Group this falcon tube and the smaller nasal **swab** together via a **rubber band**.
3. Label 1 **15-mL falcon tube** (with 3 mL DPBS) with "###### Pharyngeal Swab" label. Group this falcon tube and the larger pharyngeal **swab** together via a **rubber band**.
4. Place both assemblages into blue biohazard bag and seal closed.

RECTAL SWAB KIT

Materials:

1. 1 **blue biohazard bag**
2. 1 **swab**
3. 1 **14-mL falcon tube** (clear top)

Steps:

1. Label 1 **blue biohazard bag** with study ID label (######) and sample kit bag label (Rectal Swab).
2. Label 1 **14-mL falcon tube** with "###### Rectal Swab" label.
3. Place tube and **swab** into blue biohazard bag and seal closed.

VAGINAL SWAB KIT

Materials:

1. 1 **blue biohazard bag**
2. 1 **swab**
3. 1 **14-mL falcon tube** (clear top)

Steps:

1. Label 1 **blue biohazard bag** with study ID label (######) and sample kit bag label (Vaginal Swab).
2. Label 1 **14-mL falcon tube** with "###### Vaginal Swab" label.
3. Place tube and **swab** into blue biohazard bag and seal closed.

COVID-19 Research Study

Cohort: COVID-19-Affected (Positive, PUI, At-Risk) Pregnant Individuals

CORD BLOOD/PLACENTA KIT

Materials:

1. 1 **clear zip-lock bag**
2. 2 **small blue biohazard bags** [for cord blood tubes after collection]
3. 1 **medium-sized clear biohazard bag** [for white box (placental samples) after collection]
4. 1 **suture removal kit**
5. 1 **4x4 sponge gauze**
6. 2 **petri dishes**
7. 1 **20-cc luer lock syringe**
8. 1 **18-gauge needle**
9. 1 **"How to Collect Cord Blood & Placenta" sheet**
10. 1 **white cryobox**
11. 1 **SST** (tiger-top tube)
12. 2 **EDTA** (purple-top tube)
13. 1 **PAXgene** (liquid-filled red-top tube)
14. 4 **15-mL falcon tubes** (orange top) **with 7.5 mL RNAlater each**
15. 2 **50-mL falcon tubes** (orange top) **with 40 mL PBS each**
16. 2 **small pieces of tape**
17. 1 **large rubber band**

Steps:

1. Label 1 **clear zip-lock bag** and 1 **white box** (lid) with "###### Cord Blood / Placenta" label.
2. Label 2 **blue biohazard bags** with "###### Cord Blood" label.
3. Label 1 **medium-sized clear biohazard bag** with "###### Placenta" label.
18. Inside **clear zip-lock bag**, place **suture removal kit**, **4x4 sponge gauze**, **20-cc luer lock syringe**, **18-gauge needle**, 2 **petri dishes**, both folded **blue biohazard bags**, folded **medium-sized clear biohazard bag**, and folded "**How to Collect Cord Blood & Placenta**" **sheet**.
4. Label 1 **SST** and 2 **EDTA** with "###### Blood – M *5 mL*" label.
5. Label 1 **PAXgene** with "###### Blood – M *2.5 mL*" label.
6. Open **white box** and place blood tubes in 1st row (closest to you) in the order **SST**, **EDTA**, **PAX**, **EDTA** (from left to right).
7. Label 2 **15-mL falcon tubes** (with 7.5 mL RNAlater) with "###### Plac-F" label. Place these tubes in the 2nd row (behind the blood tubes).
8. Label 2 **15-mL falcon tubes** (with 7.5 mL RNAlater) with "###### Plac-M" label. Place these tubes in the 3rd row (behind the "Plac-F" falcons).
9. Label 2 **50-mL falcon tubes** (with 40 mL DPBS) with "DPBS Rinse (saline)" label. Place these tubes in the 4th row (behind the "Plac-M" falcons).
10. Place lid back onto **white box** (ensuring that lid label is in front), and **tape** two opposite sides.
11. Place **clear zip-lock bag** on top of **white box** and group together via a **large rubber band**.
