# Supplementary material for: Rapid establishment of a COVID-19 perinatal biorepository: early lessons from the first 100 women enrolled
Source: BMC Med Res Methodol. 2020 Aug 26;20:215. doi: 10.1186/s12874-020-01102-y (PMC7447612; doi:10.1186/s12874-020-01102-y)
Supplement: Supplementary file 8 — Additional file 8. REDCap data fields. [file 12874_2020_1102_MOESM8_ESM.pdf]

Urgent Demographics

Study ID (Ragon): ________________________________

MRN: ________________________________

Demographics:

Maternal Age: ________________________________

Maternal Race:
- ☐ White or Caucasian
- ☐ American Indian or Alaskan Native
- ☐ Black or African American
- ☐ Asian
- ☐ Native Hawaiian or Other Pacific Islander
- ☐ Other
- ☐ Unknown/Not reported

((choose all that apply))

Indicate Other: ________________________________

Maternal Ethnicity:
- ○ Hispanic or Latino
- ○ Not Hispanic or Latino
- ○ Unknown/Not Reported

Insurance Type:
- ○ Private
- ○ Public
- ○ Unknown

Maternal History:

Maternal Comorbidities:
- ☐ Chronic HTN
- ☐ Diabetes/GDM
- ☐ BMI > 30
- ☐ Asthma
- ☐ Other pre-existing pulmonary disease
- ☐ Chronic kidney disease
- ☐ HIV
- ☐ IBD (Crohn's/UC)
- ☐ Autoimmune disease
- ☐ History of transplant or immune suppressive meds
- ☐ Thyroid disease
- ☐ Cancer
- ☐ N/A

Substance Abuse:

(within past year)
- ☐ Alcohol
- ☐ Cigarettes/Tobacco
- ☐ Marijuana
- ☐ Other vape
- ☐ Opioids
- ☐ Opioid replacement therapy
- ☐ Other
- ☐ N/A

Indicate Other: __________________________________

Pregnancy History:

Gravidity:

(Include current pregnancy) __________________________________ ('N/A' if unknown)

Parity:

(Exclude current delivery) __________________________________ ('N/A' if unknown)

History of Pre-Term Birth: __________________________________ ('N/A' if unknown)

Current Pregnancy:

Pre-Pregnancy BMI (kg/m^2): __________________________________ ('N/A' if unknown)

If Pre-Pregnancy BMI Unknown, Indicate (Early) Pregnancy BMI (kg/m^2): __________________________________ ('N/A' if unknown)

Specify Gestational Age: __________________________________ (1/7=0.1, 2/7=0.3, 3/7=0.4, 4/7=0.6, 5/7=0.7, 6/7=0.9)

Pre-pregnancy BMI (kg/m^2):
- ◯ Underweight (< 18.5)
- ◯ Normal (18.5-24.9)
- ◯ Overweight (25.0-29.9)
- ◯ Obese (≥ 30.0)

Gestational Weight Gain (lbs): __________________________________ ('N/A' if unknown)

Main prenatal care site
- ◯ Community Health Clinic
- ◯ MGH Main Campus
- ◯ Other

Indicate Other __________________________________

Admission:

COVID-19 Status at Admission/Enrollment:

- ◯ Positive (symptomatic)
- ◯ Positive (asymptomatic)
- ◯ Negative (PUI screen)
- ◯ Negative (asymptomatic, universal screen)
- ◯ Negative (recovered)
- ◯ Unknown/not tested

Date of enrollment

____

Previously symptomatic?

- ◯ Yes
- ◯ No

Date of COVID-19 Positive Test:

____

Date of COVID-19 Negative Test:

____

Gestational Age at Time of Test Positive or Test Negative:

- ◯ Late term (> 40 weeks 0 days)
- ◯ Early/Full term (37 weeks 0 days - 40 weeks 0 days)
- ◯ Late preterm (34 weeks 0 days - 36 weeks 6 days)
- ◯ Moderate preterm (32 weeks 0 days - 33 weeks 6 days)
- ◯ Very preterm (28 weeks 0 days - 31 weeks 6 days)
- ◯ Extreme preterm (less than 28 weeks)

Specify Gestational Age at Test Positive/Negative:

____
(1/7=0.1, 2/7=0.3, 3/7=0.4, 4/7=0.6, 5/7=0.7, 6/7=0.9)

Date of onset of symptoms

____

Symptoms at Test Positive/Negative:

- ☐ Cough
- ☐ Fever/Chills
- ☐ Shortness of breath
- ☐ Congestion
- ☐ Loss of taste/smell
- ☐ Myalgias
- ☐ Fatigue
- ☐ Nausea/Vomiting
- ☐ Headache
- ☐ Diarrhea
- ☐ Other
- ☐ N/A

Indicate Other:

____

Other Virus Detected?

- ☐ Influenza
- ☐ RSV
- ☐ Other
- ☐ N/A

Indicate Other: __________________________________

COVID-19 or Pulmonary Therapies:
- ☐ Remdesivir
- ☐ Hydroxychloroquine
- ☐ Inhaled Nitric Oxide
- ☐ Ceftriaxone
- ☐ Azithromycin
- ☐ Other
- ☐ N/A

Blinded Remdesivir Trial?
- ☐ Yes
- ☐ No
- ☐ Unknown

Indicate Other: __________________________________

Time(s) of sample collection
- ☐ Antepartum
- ☐ Delivery
- ☐ Postpartum

Antepartum maternal blood collection date __________________________________

Antepartum urine collection date __________________________________

Antepartum stool collection date __________________________________

Antepartum saliva collection date __________________________________

Antepartum sputum collection date __________________________________

Antepartum nasal swab collection date __________________________________

Antepartum oral swab collection date __________________________________

Antepartum rectal swab collection date __________________________________

Antepartum vaginal swab collection date __________________________________

Delivery maternal blood collection date __________________________________

Delivery cord blood collection date __________________________________

Delivery placenta collection date	__________________
Delivery urine collection date	__________________
Delivery stool collection date	__________________
Delivery saliva collection date	__________________
Delivery sputum collection date	__________________
Delivery nasal swab collection date	__________________
Delivery oral swab collection date	__________________
Delivery rectal swab collection date	__________________
Delivery vaginal swab collection date	__________________
Delivery breastmilk collection date	__________________
Postpartum maternal blood collection date	__________________
Postpartum urine collection date	__________________
Postpartum stool collection date	__________________
Postpartum saliva collection date	__________________
Postpartum sputum collection date	__________________
Postpartum nasal swab collection date	__________________
Postpartum oral swab collection date	__________________
Postpartum rectal swab collection date	__________________

Postpartum vaginal swab collection date

____

Postpartum breastmilk collection date

____

Antibiotic Use

Indicate Antibiotics Taken Within 3 Months of Admission/Sample Collection:

(Home meds)

- ☐ Amoxicillin
- ☐ Azithromycin
- ☐ Ampillcin
- ☐ Penicillin
- ☐ Gentamycin
- ☐ Clindamycin
- ☐ Ampicllin/Sulbactam (Unasyn)
- ☐ Cefazolin (Ancef)
- ☐ Ceftriaxone
- ☐ Vancomycin
- ☐ Cefipime
- ☐ Metronidazole (Flagyl)
- ☐ Piperacillin/Tazobactam (Zosyn)
- ☐ Erythromycin
- ☐ Cephalexin (Keflex)
- ☐ Other
- ☐ N/A

Indicate Other:

____

Indicate Antibiotics Taken During Admission for Treatment:

(L&D or COVID-related admission)

- ☐ Amoxicillin
- ☐ Azithromycin
- ☐ Ampillcin
- ☐ Penicillin
- ☐ Gentamycin
- ☐ Clindamycin
- ☐ Ampicllin/Sulbactam (Unasyn)
- ☐ Cefazolin (Ancef)
- ☐ Ceftriaxone
- ☐ Vancomycin
- ☐ Cefipime
- ☐ Metronidazole (Flagyl)
- ☐ Piperacillin/Tazobactam (Zosyn)
- ☐ Erythromycin
- ☐ Cephalexin (Keflex)
- ☐ Other
- ☐ N/A

Indicate Other:

____

Indicate Antibiotics Taken During Admission for Prophylaxis:<br><br>(L&D admission)	☐ Amoxicillin<br>☐ Azithromycin<br>☐ Ampillcin<br>☐ Penicillin<br>☐ Gentamycin<br>☐ Clindamycin<br>☐ Ampicllin/Sulbactam (Unasyn)<br>☐ Cefazolin (Ancef)<br>☐ Ceftriaxone<br>☐ Vancomycin<br>☐ Cefipime<br>☐ Metronidazole (Flagyl)<br>☐ Piperacillin/Tazobactam (Zosyn)<br>☐ Erythromycin<br>☐ Cephalexin (Keflex)<br>☐ Other<br>☐ N/A
Indicate Other:	______________________________
Received ≥ 1 Course of Betamethasone for Fetal Lung Maturity?	○ Yes<br>○ No<br>○ Unknown

Lab Results

Lowest WBC Count (K/µL):	______________________________
Highest WBC Count (K/µL):	______________________________
Lowest Neutrophils (K/µL):	______________________________
Lowest Lymphocytes (K/µL):	______________________________
Lowest Hematocrit (%):	______________________________
Lowest Platelets (K/µL):	______________________________
Highest AST (Units/L):	______________________________
Highest ALT (Units/L):	______________________________
Highest Cr (mg/dL):	______________________________
Highest CRP (mg/L) :	______________________________

Highest D-dimer (µg/mL): ______________________

Delivery:

Maternal COVID-19 Status at Delivery:
- ◯ Positive (symptomatic)
- ◯ Positive (asymptomatic)
- ◯ Negative (PUI screen)
- ◯ Negative (asymptomatic, universal screen)
- ◯ Negative (recovered)
- ◯ Unknown/not tested

Gestational Age at Delivery:
- ◯ Late term (> 40 weeks 0 days)
- ◯ Early/Full term (37 weeks 0 days - 40 weeks 0 days)
- ◯ Late preterm (34 weeks 0 days - 36 weeks 6 days)
- ◯ Moderate preterm (32 weeks 0 days - 33 weeks 6 days)
- ◯ Very preterm (28 weeks 0 days - 31 weeks 6 days)
- ◯ Extreme preterm (less than 28 weeks)

Specify Gestational Age at Delivery: ______________________
(1/7=0.1, 2/7=0.3, 3/7=0.4, 4/7=0.6, 5/7=0.7, 6/7=0.9)

Number of the Current Pregnancy Gestations:
- ◯ Singleton
- ◯ Twin
- ◯ Triplet
- ◯ Other

Indicate Other: ______________________

Any Labor?
- ◯ Yes
- ◯ No

Delivery Mode:
- ◯ Vaginal delivery
- ◯ Cesarean section

CS Priority:
- ◯ Unscheduled (urgent/emergent)
- ◯ Scheduled

If CS [check all that apply]
- ☐ Arrest indication (cephalopelvic disproportion, arrest of dilation, arrest of descent, active phase arrest, failed induction)
- ☐ Fetal indication (nonreassuring fetal heart tracing, fetal distress)
- ☐ Breech
- ☐ Placenta Previa
- ☐ Multiple gestation
- ☐ Elective Repeat
- ☐ Other

Indicate Other: ______________________

Preeclampsia or Gestational HTN?
- ◯ Yes
- ◯ No

Composite Maternal Morbidity:

- ☐ Hemorrhage
- ☐ Eclampsia
- ☐ ARDS (Acute Respiratory Distress Syndrome)
- ☐ DIC (Disseminated Intravascular Coagulation)
- ☐ ARF (Acute Renal Failure)
- ☐ Acute MI (Myocardial Infarction)
- ☐ AFE (Amniotic Fluid Embolism)
- ☐ CHF (Congestive Heart Failure)
- ☐ Sepsis/Shock
- ☐ Assisted Ventilation
- ☐ Hysterectomy
- ☐ ICU Admission
- ☐ N/A

Neonate 1:

Date/Time of Delivery: ______________________
(Use 24 hour clock)

MRN of Neonate 1: ______________________

Sex Assigned At Birth:

- ○ Male
- ○ Female

Birth Weight: ______________________
(Use grams, 'N/A' if unknown)

Fetal Growth Restriction?

- ○ Yes
- ○ No
- ○ Unknown

Neonate COVID-19 Screen Result:

- ○ Negative
- ○ Positive
- ○ Unknown/not tested

Apgar at 1 Minute: ______________________
('N/A' if unknown)

Apgar at 5 Minutes: ______________________
('N/A' if unknown)

Composite Infant Morbidity:

- ☐ Death
- ☐ RDS (Respiratory Distress Syndrome)
- ☐ TTN (Transient Tachypnea of the Newborn)
- ☐ NEC (Necrotizing Entercolitis)
- ☐ Sepsis
- ☐ Assisted Ventilation
- ☐ Seizure
- ☐ Grade 3/4 IVH (Intraventricular Hemorrhage)
- ☐ CPAP or Supplemental O2 (Non-Invasive Respiratory Support)
- ☐ NICU Admission
- ☐ N/A

Neonate 1 Lab Results:

Lowest WBC Count (K/μL): ______________________

Highest WBC Count (K/μL): ______________________

Lowest Neutrophils (K/μL): ______________________

Lowest Lymphocytes (K/μL): ______________________

Lowest Hematocrit (%): ______________________

Lowest Platelets (K/μL): ______________________

Highest AST (Units/L): ______________________

Highest ALT (Units/L): ______________________

Highest Cr (mg/dL): ______________________

Highest CRP (mg/L) : ______________________

Highest D-dimer (μg/mL): ______________________

Neonate 2:

Date/Time of Delivery: ______________________

MRN of Neonate 2: ______________________

Sex Assigned At Birth:
- ◯ Male
- ◯ Female

Birth Weight: ______________________
(Use grams, 'N/A' if unknown)

Fetal Growth Restriction?
- ◯ Yes
- ◯ No
- ◯ Unknown

Neonate COVID-19 Screen Result:
- ◯ Negative
- ◯ Positive
- ◯ Unknown/not tested

Apgar at 1 Minute: ____________________ ('N/A' if unknown)

Apgar at 5 Minutes: ____________________ ('N/A' if unknown)

Composite Infant Morbidity:
- ☐ Death
- ☐ RDS (Respiratory Distress Syndrome)
- ☐ TTN (Transient Tachypnea of the Newborn)
- ☐ NEC (Necrotizing Entercolitis)
- ☐ Sepsis
- ☐ Assisted Ventilation
- ☐ Seizure
- ☐ Grade 3/4 IVH (Intraventricular Hemorrhage)
- ☐ CPAP or Supplemental O2 (Non-Invasive Respiratory Support)
- ☐ NICU Admission
- ☐ N/A

Neonate 2 Lab Results:

Lowest WBC Count (K/µL): ____________________

Highest WBC Count (K/µL): ____________________

Lowest Neutrophils (K/µL): ____________________

Lowest Lymphocytes (K/µL): ____________________

Lowest Hematocrit (%): ____________________

Lowest Platelets (K/µL): ____________________

Highest AST (Units/L): ____________________

Highest ALT (Units/L): ____________________

Highest Cr (mg/dL): ____________________

Highest CRP (mg/L) : ____________________

Highest D-dimer (µg/mL): ______________________________

Notes/Comments: ______________________________
